# LcrQ Coordinates with the YopD-LcrH Complex To Repress *lcrF* Expression and Control Type III Secretion by Yersinia pseudotuberculosis

**DOI:** 10.1128/mBio.01457-21

**Published:** 2021-06-22

**Authors:** Keke Fei, Huan Yan, Xiaoyan Zeng, Shaojia Huang, Wei Tang, Matthew S. Francis, Shiyun Chen, Yangbo Hu

**Affiliations:** a CAS Key Laboratory of Special Pathogens and Biosafety, Wuhan Institute of Virology, Center for Biosafety Mega-Science, Chinese Academy of Sciences, Wuhan, China; b Department of Molecular Biology, Umeå University, Umeå, Sweden; c Umeå Centre for Microbial Research, Umeå University, Umeå, Sweden; d State Key Laboratory of Virology, Wuhan Institute of Virology, Center for Biosafety Mega-Science, Chinese Academy of Sciences, Wuhan, China; e University of Chinese Academy of Sciences, Beijing, China; UCLA School of Medicine

**Keywords:** T3SS, regulation, RNase E, RhlB, chaperone

## Abstract

Human-pathogenic *Yersinia* species employ a plasmid-encoded type III secretion system (T3SS) to negate immune cell function during infection. A critical element in this process is the coordinated regulation of T3SS gene expression, which involves both transcriptional and posttranscriptional mechanisms. LcrQ is one of the earliest identified negative regulators of *Yersinia* T3SS, but its regulatory mechanism is still unclear. In a previous study, we showed that LcrQ antagonizes the activation role played by the master transcriptional regulator LcrF. In this study, we confirm that LcrQ directly interacts with LcrH, the chaperone of YopD, to facilitate the negative regulatory role of the YopD-LcrH complex in repressing *lcrF* expression at the posttranscriptional level. Negative regulation is strictly dependent on the YopD-LcrH complex, more so than on LcrQ. The YopD-LcrH complex helps to retain cytoplasmic levels of LcrQ to facilitate the negative regulatory effect. Interestingly, RNase E and its associated protein RhlB participate in this negative regulatory loop through a direct interaction with LcrH and LcrQ. Hence, we present a negative regulatory loop that physically connects LcrQ to the posttranscriptional regulation of LcrF, and this mechanism incorporates RNase E involved in mRNA decay.

## INTRODUCTION

All three human-pathogenic *Yesinia* species—Y. pestis, Y. enterocolitica, and Y. pseudotuberculosis—employ a type III secretion system (T3SS) to deliver immunomodulatory effector proteins into host immune cells (1–4). This has the purpose to hijack cellular signaling involved in host immune responsiveness that enables bacteria to establish an infection niche (1–3). All structural proteins (termed Ysc for *Yersinia* secretion), as well as the major secreted effectors (termed Yops for *Yersinia* outer proteins), are encoded on a 70-kb conserved virulence plasmid named pYV or pCD. Additionally, a recent report also indicates a subset of immunomodulatory effector proteins are encoded on the *Yersinia* chromosome (5).

Composed of several highly conserved substructures, both T3SS biogenesis and subsequent substrate secretion follow well-orchestrated pathways that are tightly controlled (6–9). In *Yersinia*, *ysc* and *yop* gene expression is stringently controlled at both transcriptional and posttranscriptional levels (7, 10–16). A low Ca^2+^ signal *in vitro* or close eukaryotic cell contact *in vivo* are both stimulators of Ysc-Yop T3SS biogenesis and activity (17, 18). LcrF, the only characterized activator encoded on the pYV plasmid, is an AraC family transcriptional regulator that directly binds to several promoters of T3SS-related genes to activate their transcription (16, 19, 20). Additionally, several pYV-encoded proteins counter this by repressing the T3SS system (21, 22). One such protein is dual-functional YopD, a translocon pore former located at the top of the T3SS needle that also acts as a negative regulator by binding to AU-rich sequences in the 5′ untranslated region (5′ UTR) of *yop* mRNA to regulate its stability and the translation processes (23–25). Crucially, a further role of YopD is to impact the effectiveness of the translational regulator CsrA, which, in turn, enhances LcrF production (26). Central to the multiple functions of YopD is the need for presecretory stabilization through a binary interaction with the cognate type III secretion (T3S) chaperone, LcrH (22, 23, 25, 27, 28).

Additionally, LcrQ, also known as YscM in Y. enterocolitica, has long been known to block Yop secretion when accumulated in the bacterial cytoplasm (17, 21, 29). However, the mechanism underlying this blockage has remained elusive. LcrQ shares 42% identity to the first 128 residues of the T3SS effector YopH (21). This explains why both LcrQ and YopH share a T3S chaperone, SycH (30–32). The derepression of *yop* expression is relieved once SycH interacts with LcrQ/YscM (33). This interaction also facilitates the type III secretion of LcrQ to the outside environment, which further elevates Yop synthesis and secretion (31). In fact, fusion of glutathione *S*-transferase (GST) tag to LcrQ protein, disruption of the T3SS apparatus, or deletion of SycH, all of which prevent LcrQ secretion, lead to decreased expression and secretion of Yops (31, 33). Hence, retention in the bacterial cytoplasm is coupled with the negative regulatory role of LcrQ.

LcrQ lacks any obvious DNA or RNA binding motifs (21, 23, 31). This is consistent with the inability to detect a specific association between LcrQ/YscM and *yop* mRNA (23, 24). These data suggested a novel mechanism of LcrQ-mediated T3SS inhibition. An initial model posits that YopD association with a small subpopulation of 30S ribosomal particles enables LcrQ/YscM to block *yop* mRNA translation (27). However, it remains unclear how this mechanism would actually result in the specific inhibition of *yop* mRNA translation.

Our previous study showed that LcrQ shared regulatory targets with the master regulator LcrF and the relative levels of these two proteins controlled T3SS synthesis (34). We failed to observe a direct protein-protein interaction between LcrF and LcrQ (34), questioning, at the time, how these two regulators might counterbalance each other to regulate T3SS. With a view to understand this process, the present study reports on an interaction between intracellular LcrQ and the T3S chaperone LcrH. We characterized this interaction in the context of repressing LcrF levels by the YopD-LcrH complex during bacterial growth under T3SS restrictive conditions. We also demonstrate that YopD abrogates the secretion of LcrQ. These observations provide a molecular basis for how LcrQ exerts a negative regulatory role on *Yersinia* T3SS.

## RESULTS

### Cytoplasmic-located LcrQ downregulates the promoter activities of *yop* genes.

**FIG 1:**
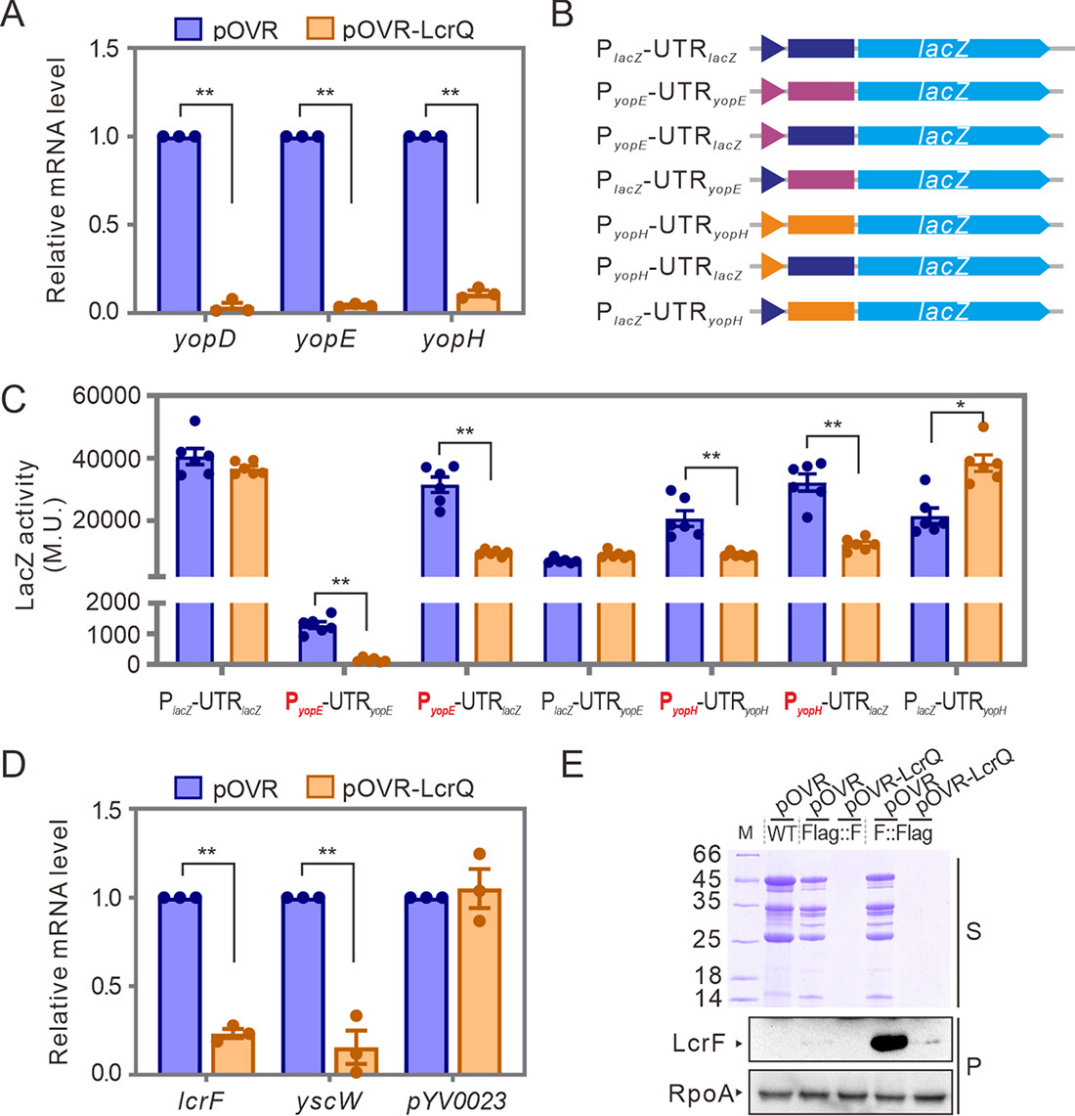
LcrQ inhibits promoter activities of *yop* genes by repressing expression of the master regulator LcrF. (A) Relative mRNA levels of *yopD*, *yopE*, and *yopH* in LcrQ-overexpressed strain. The mRNA levels in YpIII strain carrying the pOVR plasmid were normalized to 1, respectively. (B) Schematic of *lacZ* fusion constructs. Promoters and 5′ UTR from different genes are colored differently. (C) Effects of overexpressed LcrQ on activity of *lacZ* fusion constructs shown in panel B. The LacZ activity is indicated by Miller unit (M.U.) from β-galactosidase activity assay. (D) Effects of LcrQ overexpression on mRNA level of *lcrF*. The *yscW*, which is cotranscribed with *lcrF*, was also tested. The *pYV0023* gene was used as a control. (E) Repression of LcrQ to expression of LcrF protein. The Flag tag was fused to either the N terminus (Flag::F) or C terminus (F::Flag) of LcrF protein in the coding region. Expression level of LcrF protein was detected by anti-Flag antibody. RpoA was detected as a loading control. *, *P* < 0.05; **, *P* < 0.01.

To confirm the negative regulatory role of LcrQ protein, we first detected the mRNA levels of *yopD*, *yopE*, and *yopH* genes in YpIII parental strain overexpressing *lcrQ*. Elevated cytoplasmic LcrQ abrogated mRNA levels of these genes under T3SS-induced conditions (Fig. 1A), which corroborated other reports (34, 35). We next aimed to identify the regulatory element targeted by LcrQ. For this purpose, we used a transcriptional fusion assay. We constructed a series of chimeric clones composed of the promoter alone, the 5′ UTR alone, or both promoter and 5′ UTR of *yopE* and *yopH* genes in front of the promoterless *lacZ* reporter (Fig. 1B). Where the endogenous regulatory element was lacking, it was substituted by the equivalent element from the regulatory sequences of the *lac* operon (Fig. 1B). As shown in Fig. 1C, LcrQ did not repress the β-galactosidase activities of clones carrying the *lac* promoter fused with 5′ UTR of *yopE* or *yopH* genes but significantly repressed the clones carrying promoters of *yopE* or *yopH* genes. Although we could not exclude the possibility that LcrQ may regulate expression of *yopE* and *yopH* through other regions (such as the coding region or 3′ UTR), our data suggest that LcrQ can downregulate Yops expression by repressing the promoter activities of *yop* genes.

### LcrQ represses the expression of the master transcriptional regulator LcrF.

Since LcrF is the only transcriptional activator of the Ysc-Yop T3SS encoded on the pYV plasmid (16), we next asked if LcrQ could regulate the expression of *lcrF*. We first detected the mRNA levels of *lcrF* in LcrQ-overexpressed and Δ*lcrQ* strains. As expected, the mRNA level of *lcrF* was increased in a Δ*lcrQ* strain under T3SS-inducible conditions (Fig. S1A in the supplemental material), while it was repressed when LcrQ was overexpressed in the YpIII parental strain (Fig. 1D). To confirm this regulatory effect, we examined the LcrF protein levels by Western blotting in this strain overexpressing LcrQ. To facilitate LcrF detection, we inserted a Flag tag-encoding fragment at the 5′ and 3′ termini within the *lcrF* gene in *cis* in the YpIII genome. The transcription of *lcrF* mRNA was only slightly influenced by inserting this Flag tag at either end (Fig. S1C). However, the Flag::LcrF was barely detectable in Western blot assay using anti-Flag antibody (Fig. 1E), probably due to alterations in protein conformation or protein stability induced by the tag. Regardless, overexpression of LcrQ in these strains repressed the expression of recombinant LcrF in both Flag::LcrF and LcrF::Flag strains, which consequently abrogated T3SS production (Fig. 1E). These data taken all together confirmed that LcrQ downregulates the production of LcrF.

10.1128/mBio.01457-21.1 FIG S1 LcrQ-mediated regulation of T3SS-associated genes. (A) Relative mRNA levels of the *lcrF* gene in YpIII WT and Δ*lcrQ* strains. The mRNA level of *lcrF* in WT strain was normalized to 1. (B) Relative mRNA levels of *yscW* and *yopE* in Δ*yopD* and Δ*lcrH* strains carrying the pOVR or pOVR-LcrQ plasmids. Each of these two genes in strains carrying pOVR plasmid was normalized to 1, respectively. (C) The mRNA level of *lcrF* in YpIII WT and strains with fusion of N terminus (Flag::F) or C terminus (F::Flag) of LcrF protein. Download FIG S1, TIF file, 3.0 MB. Copyright © 2021 Fei et al. 2021 Fei et al. https://creativecommons.org/licenses/by/4.0/ This content is distributed under the terms of the Creative Commons Attribution 4.0 International license.

### The negative regulatory role of LcrQ is dependent on a YopD-LcrH complex.

**FIG 2:**
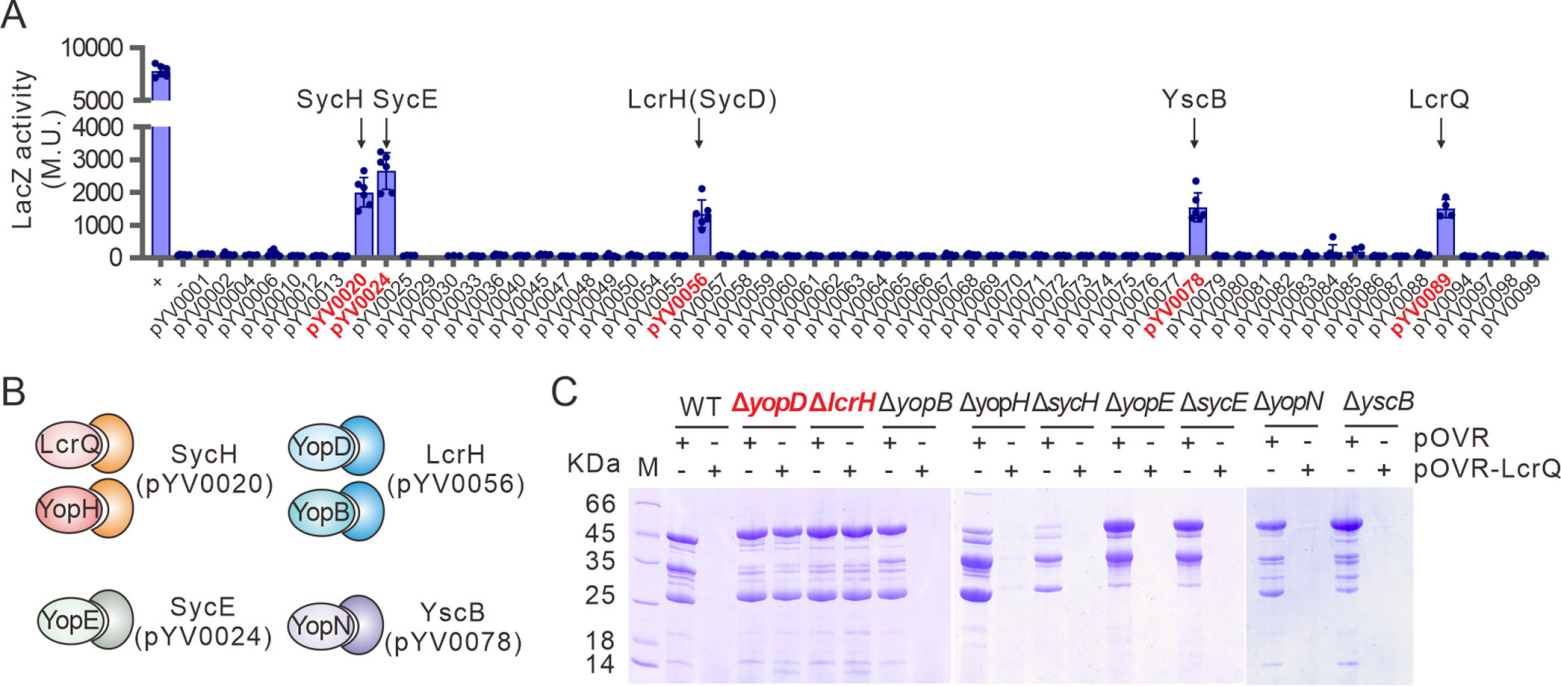
Negative regulatory role of LcrQ to LcrF is dependent on the presence of YopD/LcrH complex. (A) Screening of LcrQ-interacting *Yersinia* T3SS proteins. Bacterial adenylate cyclase two-hybrid system was applied in protein-protein interaction screening. Gene locus numbers of proteins that showed positive interaction with LcrQ are indicated in red. (B) Pairs of Yop effectors and their chaperones. (C) Effects of overexpressed LcrQ on Yops secretion in YpIII WT or mutants lacking a Yop-encoding gene (Δ*yopD*, Δ*yopB*, Δ*yopH*, Δ*yopE*, or Δ*yopN*) or their associated chaperone-encoding gene (Δ*lcrH*, Δ*sycH*, Δ*sycE*, or Δ*yscB*).

Previous analyses have shown that LcrQ does not contain any DNA or RNA binding motif (21, 23, 31). Our recent study also indicated that LcrQ does not directly interact with LcrF (34). Therefore, we suppose that LcrQ may downregulate LcrF expression by interacting with other proteins. To test this hypothesis, we screened proteins interacting with LcrQ using a bacterial two-hybrid system configured to contain a library of about 60 *ysc-yop* T3SS functional genes derived from the pYV plasmid but excluding genes involved in plasmid replication. Interestingly, LcrQ interacted with itself (Fig. 2A). Additionally, LcrQ interacted with SycH (pYV0020), SycE (pYV0024), LcrH (also known as SycD, pYV0056), and YscB (pYV0078) (Fig. 2A and B), which are customized T3S chaperones specific to the secreted Yops.

**FIG 3:**
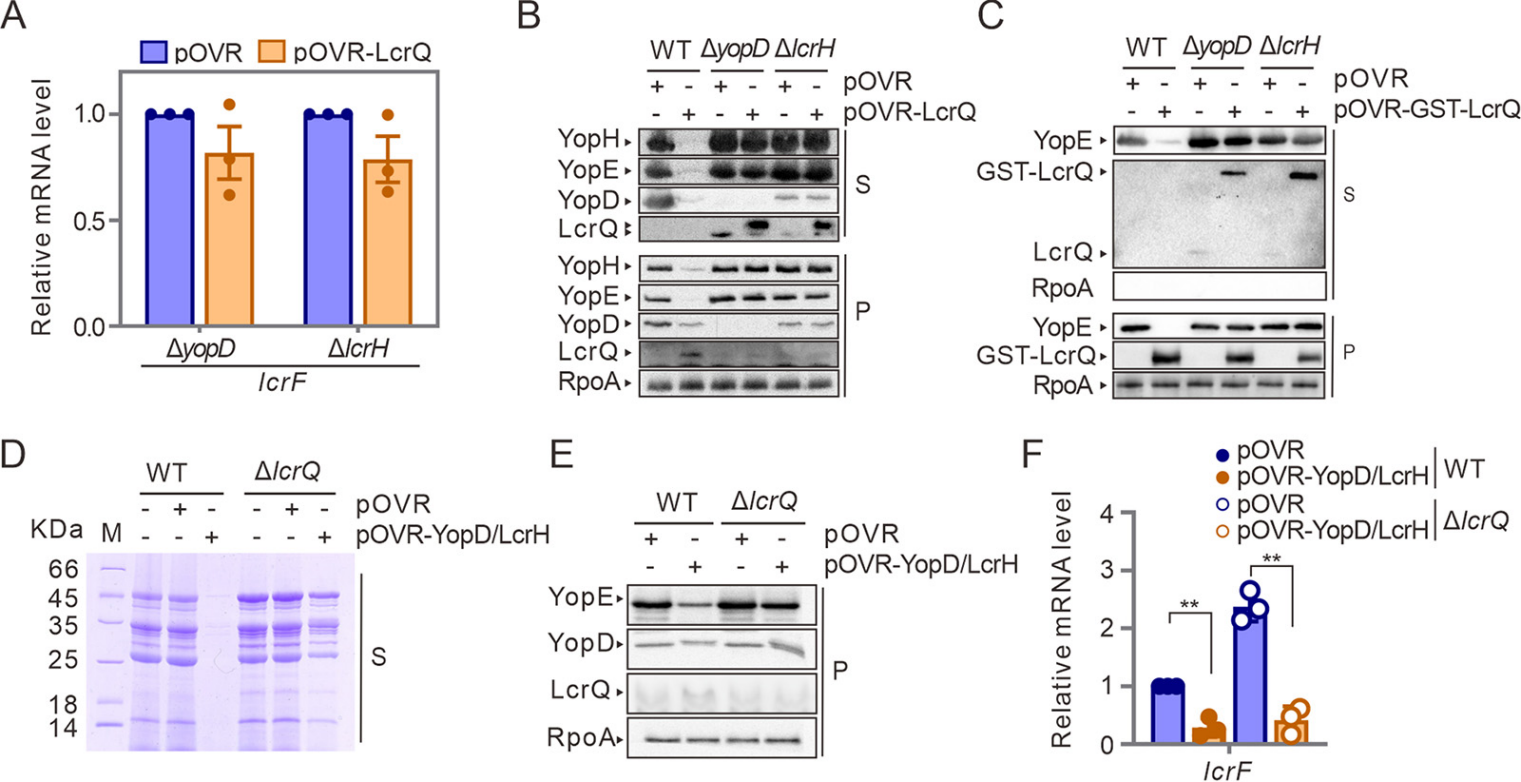
LcrQ coordinates with YopD/LcrH complex in repressing T3SS. (A) Effects of LcrQ overexpression on mRNA levels of *lcrF* in Δ*yopD* and Δ*lcrH* strains. The mRNA levels of *lcrF* in YpIII strains carrying pOVR plasmid were normalized to 1, respectively. (B) Effects of overexpressed LcrQ on Yops expression in cell pellets (P) and protein secretion (S) in YpIII WT, Δ*yopD*, and Δ*lcrH* strains. The T3SS-related proteins were detected using protein-specific antiserum. (C) Expression and secretion of Yops and LcrQ in GST-LcrQ-overexpressed strains. RpoA in supernatant was detected to exclude the possibility of contamination of cell lyses faction. (D and E) Repressive effects of overexpressed YopD/LcrH complex on Yops secretion (D) and Yops expression (E) in YpIII WT and Δ*lcrQ* strains. (F) Overexpression of YopD/LcrH complex on *lcrF* mRNA level in YpIII WT and Δ*lcrQ* strains. The *lcrF* mRNA level in WT strain carrying pOVR was normalized to 1. **, *P* < 0.01.

To understand the relevance of LcrQ-T3S chaperone interactions, we first overexpressed LcrQ in mutants lacking these T3S chaperones or their cognate Yop substrate. As shown in Fig. 2C, only deletion mutations of *yopD* (designated Δ*yopD*) or *lcrH* genes (Δ*lcrH*) abolished the downregulation function by LcrQ. Moreover, overexpression of LcrQ in the absence of YopD or LcrH could not inhibit the accumulation of *lcrF*-, *yscW*-, and *yopE*-specific mRNA (Fig. 3A and Fig. S1B). This suggests that the negative regulatory role of LcrQ depends upon the presence of functional YopD and LcrH.

During this analysis, it became evident that the intracellular level of LcrQ was much lower when overexpressed in the Δ*yopD* or Δ*lcrH* background than the wild-type (WT) background (Fig. 3B). Consistent with this, a large portion of LcrQ was secreted into the supernatants of these mutants (Fig. 3B). Hence, it appears that a YopD-LcrH complex may inhibit LcrQ secretion. To explore this relationship, we appended the GST tag to the N terminus of LcrQ, which had been observed to abolish the secretion of YscM (a LcrQ homologue) (31). Surprisingly, a portion of GST-LcrQ was observed in the clear supernatant fractions of the Δ*yopD* or Δ*lcrH* strain, although not by the parental strain that contained functional YopD and LcrH (Fig. 3C). This is likely to be active secretion to the culture supernatant rather than by contamination of bacterial cellular material because cytoplasmic-located RpoA was not detected in our supernatant samples (Fig. 3C). Critically, GST-LcrQ trapped in the cytoplasm of the Δ*yopD* or Δ*lcrH* strain had no repressive effect on YopE synthesis (Fig. 3C), although it does repress both expression and secretion of YopE when overexpressed in the YpIII parental strain (34). Together, these data suggest that intracellular LcrQ functions through the YopD-LcrH complex, and this complex retains LcrQ in the bacterial cytoplasm.

Since intracellular LcrQ requires the presence of the YopD-LcrH complex for its negative regulatory role, we next tested if the repressive effect of intracellular YopD/LcrH requires the presence of LcrQ. Noticeably, overexpression of YopD and LcrH only slightly repressed Yops secretion and synthesis in a Δ*lcrQ* strain, whereas it caused a dramatic repression in the YpIII parental background (Fig. 3D and E). On the other hand, *lcrF*-specific mRNA was repressed in both the parental and the Δ*lcrQ* backgrounds upon YopD/LcrH overexpression (Fig. 3F). These data suggest that both LcrQ-dependent and independent pathways can promote the repressive effects of YopD-LcrH.

### Mapping regulatory regions within LcrQ.

**FIG 4:**
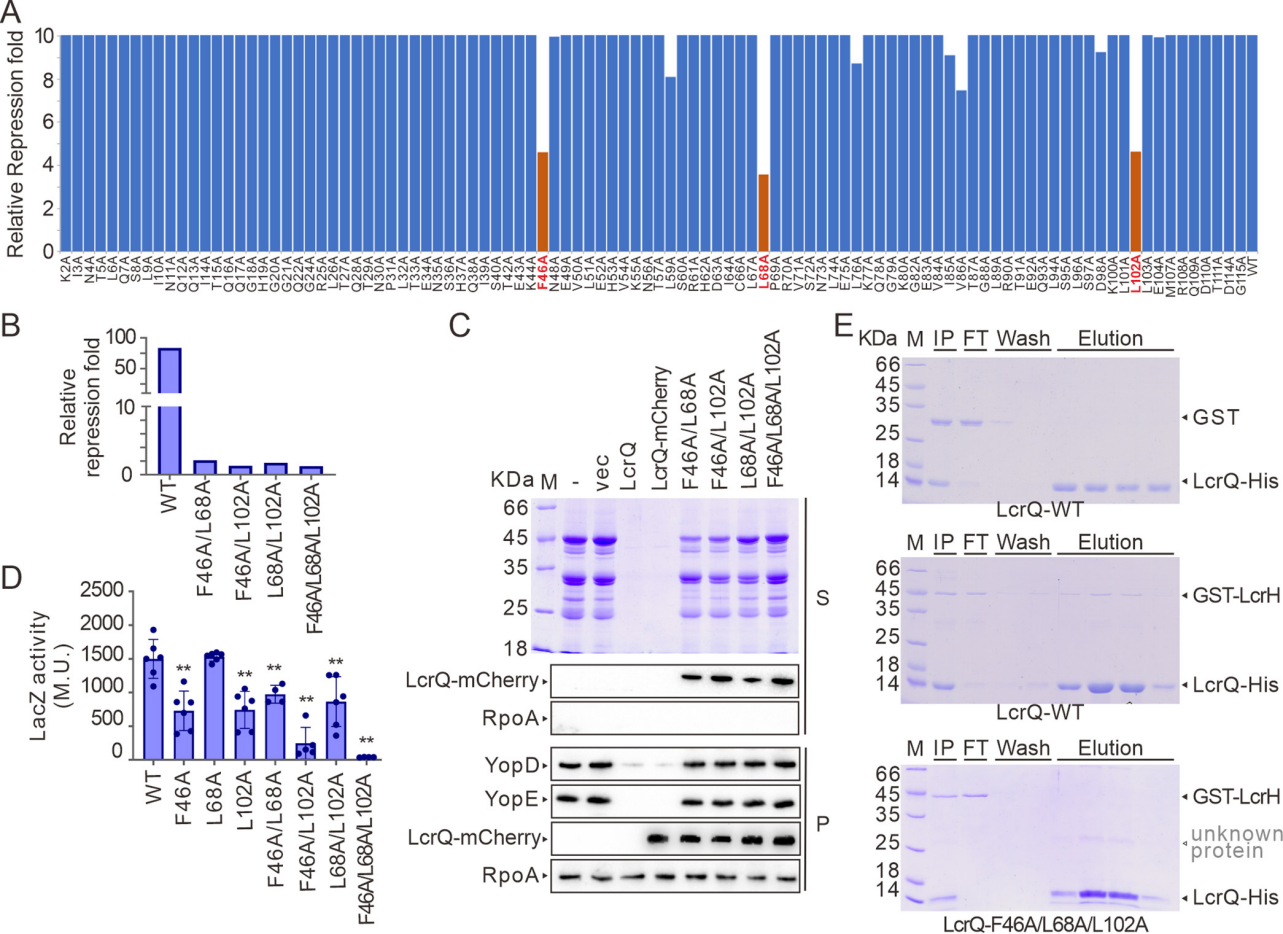
Residues F46, L68, and L102 are important for the negative regulatory role of LcrQ. (A) Scanning mutagenesis of *lcrQ* and correlation to diminished repression by the corresponding mutated product. The relative repression fold was calculated using the repression fold of LcrQ to *lcrG* promoter activity against the LcrQ protein level, which was monitored using mCherry fluorescence intensity. Data are average of three colonies. Mutations showed decreased repressive effects are indicated in red. (B and C) Measuring the effects of combinatory double or triple point mutations within *lcrQ* on the ability of LcrQ to repress *lcrG* promoter activity (B) and Yops expression and secretion (C). (D) Measuring the effects of point mutations within *lcrQ* on the ability of LcrQ to interact with LcrH in bacterial two-hybrid assays. **, *P* < 0.01. (E) Interaction of LcrQ WT or triple mutations (containing His tag) with GST-LcrH protein in pulldown assay using Ni-NTA. GST protein was used as a control.

In the absence of any predicted structural elements within LcrQ, we wanted to define regions that were important for its regulatory role. To facilitate this, we constructed an LcrQ-mCherry mutant library whereby 102 of 115 LcrQ residues were substituted for alanine. The remaining 12 preexisting alanine residues and the methionine initiation codon were left unchanged. Fusion to mCherry enabled convenient monitoring of the recombinant LcrQ mutant expression level. A biosensor assay based upon the *lcrG* promoter transcriptionally fused to promoterless *lacZ* was established as a screen for the repressive effect of LcrQ on T3SS expression. The repressive effect was determined by calculating the ratio of the fold repression relative to the respective LcrQ mutant expression level. As seen in Fig. 4A and Table S1, the relative repression fold of the three mutants, LcrQ_F46A_, LcrQ_L68A_, and LcrQ_L102A_, was considerably lower than observed for all other variants, including wild-type LcrQ. Hence, these three residues are important for the full repressive function of LcrQ. Interestingly, no single mutant totally abolished the repressive role of LcrQ (Fig. 4A). As a consequence, we constructed the F46A, L68A, and L102A mutations in double and triple combinations. This generated stable LcrQ variants with far greater regulatory defects, with the triple mutation combination, LcrQ_F46A, L68A, L102A_, being particularly defective (Fig. 4B). As expected, ectopic overexpression of this stable LcrQ_F46A, L68A, L102A_ variant failed to repress the accumulation of *lcrF-* and *yopE-*specific mRNA levels (Fig. S2) and the synthesis and secretion of Yops (Fig. 4C) under T3SS-permissive conditions. Hence, this scanning mutagenesis approach has identified crucial LcrQ residues that support its negative regulatory role.

10.1128/mBio.01457-21.2 FIG S2 Mutations with *lcrQ* affect control of *Yersinia* T3SS. Relative mRNA levels of *lcrF* and *yopE* in YpIII strains overexpressing a wild-type (LcrQ-mCherry) or mutated form of LcrQ (F46A/L68A/L102A). The mRNA level in the strain carrying the empty vector (vec) was normalized to 1. Download FIG S2, TIF file, 1.3 MB. Copyright © 2021 Fei et al. 2021 Fei et al. https://creativecommons.org/licenses/by/4.0/ This content is distributed under the terms of the Creative Commons Attribution 4.0 International license.

10.1128/mBio.01457-21.5 TABLE S1 Expression levels of LcrQ single-point mutants and their repression effects to the *lcrG* promoter activities. Download Table S1, DOCX file, 0.03 MB. Copyright © 2021 Fei et al. 2021 Fei et al. https://creativecommons.org/licenses/by/4.0/ This content is distributed under the terms of the Creative Commons Attribution 4.0 International license.

Having identified LcrH as a novel regulatory target of LcrQ, we next examined if the single, double, and triple mutant combinations of LcrQ influenced the interaction with LcrH. Initially using the bacterial two-hybrid system, we found that LcrQ_L68A_ maintained an ability to engage with LcrH to a level observed for wild-type LcrQ (Fig. 4D). On the other hand, the single (LcrQ_F46A_ and LcrQ_L102A_) and double (LcrQ_F46A, L68A_, and LcrQ_L68A, L102A_) mutant variants decreased the LcrQ-LcrH interaction as judged by a 2- to 3-fold reduction in reporter output (Fig. 4D). Furthermore, the double (LcrQ_F46A, L102A_) and triple (LcrQ_F46A, L68A, 102A_) mutation variants abrogated much of the interaction with LcrH (Fig. 4D). Critically, this was not due to protein instability because the fluorescence intensity of LcrQ_F46A, L102A_ and LcrQ_F46A, L68A, 102A_ in fusion with mCherry was comparable to wild-type LcrQ (Table S2). To further confirm these findings, we established a pulldown assay using strains producing His-tagged LcrQ variants together with either GST alone or a GST-LcrH fusion. GST-LcrH could be successfully coeluted with wild-type His-LcrQ but not with the His-LcrQ_F46A, L68A, L102A_ variant (Fig. 4E). Crucially, GST alone did not coelute with either His-LcrQ variant (Fig. 4E). Moreover, neither GST-LcrH nor GST alone could bind to Ni-nitrilotriacetic acid (Ni-NTA) in the absence of His-LcrQ (Fig. S3). Taken all together, these data suggest that the residues at positions 46 and 102 are critical for interacting with LcrH, and this interaction permits LcrQ to exert a negative regulatory role. Intriguingly, we also identified position 68 to influence this LcrQ regulatory capacity, but this may occur independently of the LcrQ-LcrH pathway.

10.1128/mBio.01457-21.3 FIG S3 Purified GST-LcrH and GST could not bind to Ni-NTA in the absence of LcrQ protein. Download FIG S3, TIF file, 0.8 MB. Copyright © 2021 Fei et al. 2021 Fei et al. https://creativecommons.org/licenses/by/4.0/ This content is distributed under the terms of the Creative Commons Attribution 4.0 International license.

10.1128/mBio.01457-21.6 TABLE S2 Expression levels of LcrQ double or triple mutants and their repression effects to the *lcrG* promoter activities. Download Table S2, DOCX file, 0.02 MB. Copyright © 2021 Fei et al. 2021 Fei et al. https://creativecommons.org/licenses/by/4.0/ This content is distributed under the terms of the Creative Commons Attribution 4.0 International license.

### RNase E contributes to negative regulation of LcrF through LcrQ and YopD-LcrH interactions.

**FIG 5:**
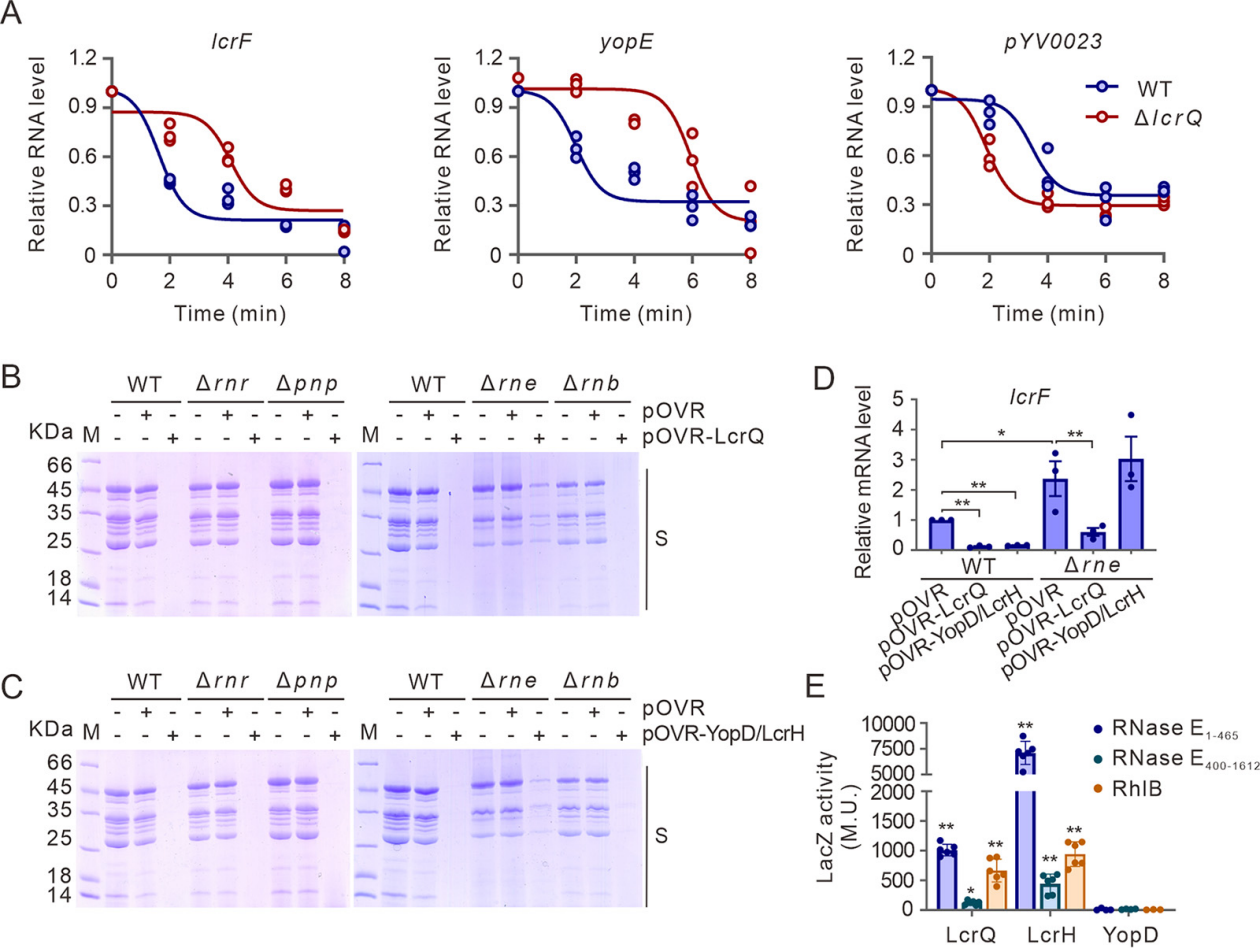
RNase E participates in the negative regulation of LcrF by LcrQ/YopD/LcrH complex. (A) mRNA stability of *lcrF*, *yopE*, and *pYV0023* in YpIII WT and Δ*lcrQ* strains. (B and C) Effects of overexpressed LcrQ (B) or YopD/LcrH complex (C) on Yops secretion in different RNase mutants (Δ*rnr*, Δ*pnp*, Δ*rne*, or Δ*rnb*). (D) *lcrF* mRNA levels in YpIII or Δ*rne* with overexpression of LcrQ or the YopD/LcrH complex. (E) Interaction of LcrQ, LcrH, or YopD with RNase E and RhlB proteins in a bacterial two-hybrid assay. RNase E was separated into two fragments, RNase E_1-465_ and RNase E_400-1612_, in this assay. *, *P* < 0.05; **, *P* < 0.01.

Since LcrQ cooperates with YopD-LcrH complex and the YopD-LcrH complex regulates T3SS posttranscriptionally (21, 23), we next tested if LcrQ also participates in posttranscriptional regulation. Consistent with our hypothesis, deletion of *lcrQ* increased the stability of *lcrF-* and *yopE-*specific mRNA, but not mRNA of the control fragment pYV0023 encoding a likely transposase remnant (Fig. 5A). To examine whether RNA decay factors are also involved in this negative regulatory circuit, we overexpressed LcrQ and YopD with LcrH in four different RNase mutant strains, Δ*rne*, Δ*pnp*, Δ*rnr,* and Δ*rnb* (36). As seen in Fig. 5B and C, the repressive impact on Yops secretion normally caused by accumulation of either LcrQ or the YopD-LcrH was diminished specifically in the Δ*rne* strain lacking RNase E production. This correlated with the observation that *lcrF-*specific mRNA was higher in this mutant than the WT strain (Fig. 5D). Crucially, overexpression of LcrQ in the Δ*rne* strain was less effective at repressing *lcrF* mRNA levels (4-fold reduction) than in the WT strain (9-fold) (Fig. 5D). Moreover, YopD/LcrH overexpression in the Δ*rne* strain had no repressive impact on *lcrF*-specific mRNA levels compared to the WT strain (Fig. 5D). Hence, the RNase E mRNA decay factor influences the negative role of LcrQ and YopD-LcrH complex.

We wondered if this association was through a direct interaction between these proteins. Using a bacterial two-hybrid system assay, we found that YopD did not show any direct interaction with RNase E, but LcrQ and LcrH can both interact with RNase E and its associated protein RhlB (Fig. 5E). Importantly, the regulatory-deficient LcrQ mutants F46A, L68A and L102A, in either single, double, or triple combination, could all still interact with RNase E or RhlB (Fig. S4). Hence, RNase E or RhlB do not compete with LcrH for the same binding sites on LcrQ. Taken altogether, these data indicate that RNase E is an important contributor to Ysc-Yop T3SS downregulation by LcrQ and YopD-LcrH control in pathogenic *Yersinia*. Further, it is likely that RNase E works through interactions with LcrQ and LcrH.

10.1128/mBio.01457-21.4 FIG S4 Measuring the effects of point mutations within *lcrQ* on the ability of LcrQ to interact with RNase E_1-456_ and its associated RhlB protein in bacterial two-hybrid assays. Download FIG S4, TIF file, 2.6 MB. Copyright © 2021 Fei et al. 2021 Fei et al. https://creativecommons.org/licenses/by/4.0/ This content is distributed under the terms of the Creative Commons Attribution 4.0 International license.

## DISCUSSION

A number of studies have highlighted the important regulatory role played by LcrQ in the control of Ysc-Yop T3SS by *Yersinia* (21, 31, 34). However, detailed knowledge of the molecular mechanism is lacking. In this study, we demonstrated that LcrQ inhibits expression of *yop* genes by downregulating the expression of *lcrF* encoding the master transcriptional regulator LcrF. This regulatory process depends on the presence of a posttranscriptional regulatory complex composed of YopD and LcrH. Furthermore, we demonstrated that coupling between LcrQ and this complex is achieved through a direct interaction of LcrQ with LcrH. Finally, these two proteins can both interact with RNase E, suggesting LcrQ, YopD/LcrH, and RNase E may combine to regulate T3SS in *Yersinia*.

**FIG 6:**
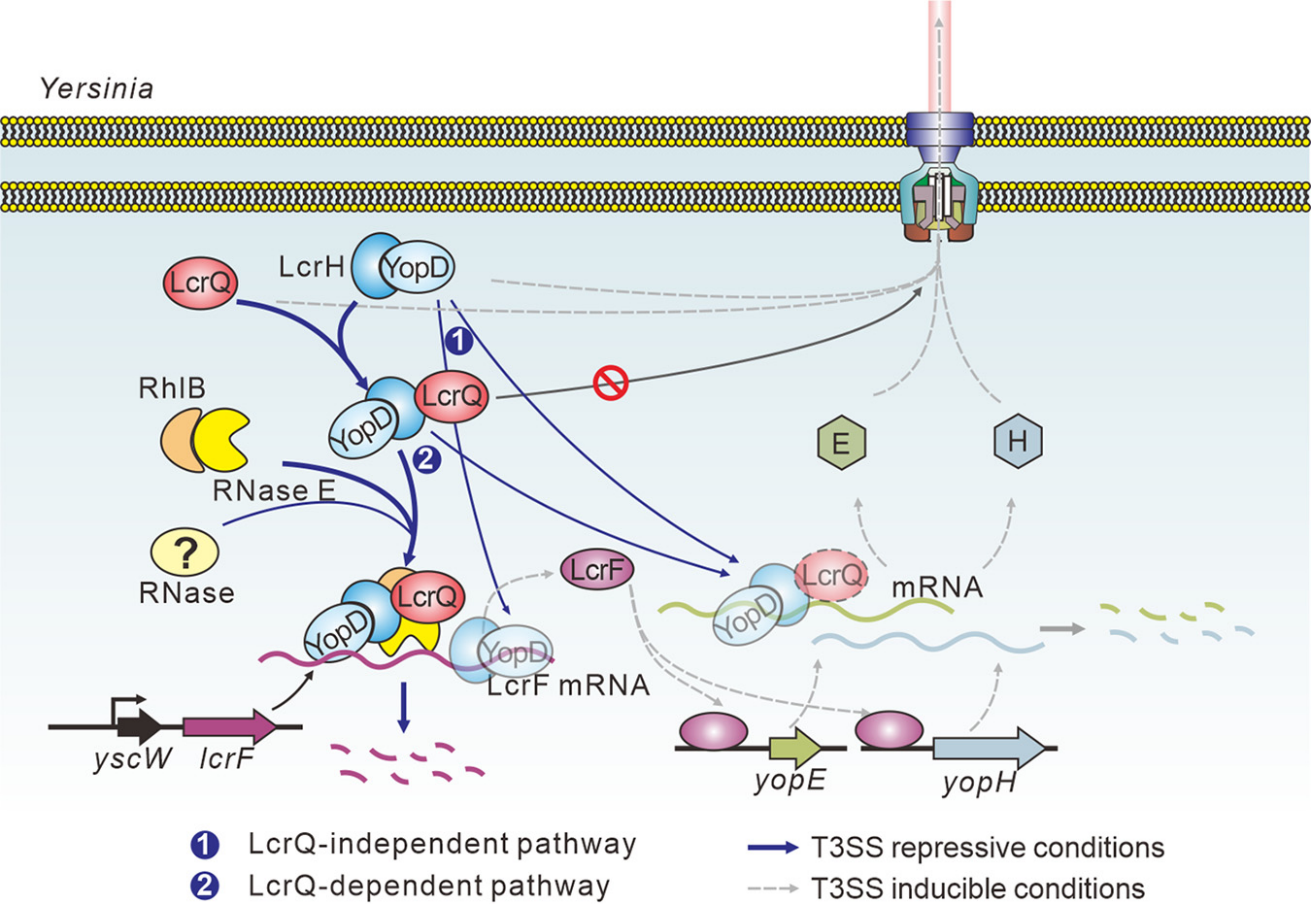
Proposed model for the role of LcrQ in regulating *Yersinia* T3SS. Under T3SS-inducible conditions, the master regulator LcrF activates the transcription of *yop* genes. The synthesized Yop proteins are then secreted outside *Yersinia* cells through the T3SS (indicated by dotted lines). Under T3SS-repressive conditions, the intracellular YopD-LcrH complex represses the expression of T3SS genes via a pathway that is either independent of LcrQ (1) or dependent on LcrQ (2). The LcrQ-dependent pathway also involves RNase E and its associated protein RhlB and possibly some other uncharacterized RNases. This involvement occurs via direct protein-protein interactions involving LcrQ with LcrH as well as LcrQ/LcrH with RNase E and RhlB. Importantly, the interaction between YopD-LcrH with LcrQ inhibits the secretion of LcrQ (🛇). LcrQ trapped in the cytoplasm subsequently promotes the repressive effect of the YopD-LcrH-LcrQ complex in a feedback pathway.

Previous studies had indicated that the negative regulatory role of LcrQ may require the presence of the YopD-LcrH complex (22, 35), but no direct mechanism underlying this possible relationship had been demonstrated experimentally. Moreover, additional studies using an *in vitro* translation system demonstrated that YopQ translation repression by the YopD-LcrH complex required the LcrQ homologue, YscM1 (13, 27). Herein, we bridge all these studies by identifying that LcrQ interacts with LcrH to facilitate the negative regulatory role of the YopD-LcrH complex. Critically, stable LcrQ variants unable to physically interact with LcrH could no longer exert a repressive role on the T3SS. These findings are supported by the observation that YscM interacts with LcrH in Y. enterocolitica (37, 38). We speculate that the purpose of this interaction might be to influence mRNA stability. The basis for this idea stems from observing that both LcrH and LcrQ interact with RNase E and its associated protein RhlB. We propose a model that suggests this interaction facilitates *lcrF* mRNA degradation (Fig. 6). Our future experiments will strive to confirm this coupling. Interestingly, previous studies with YopD have indicated a role in mRNA stability (24–26). In fact, the recent work of Kusmierek and colleagues indicates that this process involves an intricate array of RNA binding proteins and degradation factors (26). Our work corroborates and extends these findings by suggesting that the mRNA stability function attributed to YopD may actually depend upon LcrQ-LcrH, which acts as a molecular scaffold to recruit RNase E in the vicinity of YopD (Fig. 6).

RNase E, which recognizes a specific AU-rich RNA motif (39, 40), is an established regulator of T3SSs in different bacteria. However, the effects can be either repression, such as in *Yersinia* (26, 41) and enterohemorrhagic Escherichia coli (EHEC) (42, 43), or activation such as with Pseudomonas aeruginosa (44). There remains a lack of detail surrounding the action of RNase E in these different modes of regulation; to fill these knowledge gaps is worthy of further studies. Our data indicate that RNase E is an important contributor to Ysc-Yop T3SS downregulation by LcrQ and YopD-LcrH control in pathogenic *Yersinia.* However, we also observed that the repressive effects of LcrQ and YopD/LcrH were not completely abolished in our Δ*rne* strain (Fig. 5). This is not so surprising given the multifactorial nature of RNase E function. For example, the basis of our Δ*rne* strain is an incomplete deletion caused by a 3′ truncation of the *rne* gene (36). It is evident that the nature of the *rne* mutation, coupled to the expression of other RNases in the organism, can affect the phenotypes displayed by *rne* mutants with respect to RNA degradosome assembly, mRNA turnover, maturation of rRNA and tRNA precursors, processing and degradation of regulatory RNAs, as well as rRNA quality control (45). Any of these situations may be at play in our *Yersinia* Δ*rne* background. Moreover, unidentified factors, such as additional RNA binding proteins, may also be involved in the regulatory roles of LcrQ and YopD/LcrH. Hence, further studies of our Δ*rne* mutant will likely identify additional players in the posttranscriptional regulation of *lcrF* expression and its impact on T3SS control by pathogenic *Yersinia*.

Interestingly, others implicate one other RNA stability factor, PNPase, in the control of T3SS in *Yersinia* (46, 47). In particular, secretion of YopE and YopD were inhibited in the absence of PNPase, but only upon a short exposure of bacteria to T3SS-inducing conditions (47). Intriguingly, prolonged exposure did not result in any defect, and this is consistent with our data (Fig. 5). Subsequently, however, PNPase was found to posttranscriptionally regulate *lcrF* expression through YopD (26). Yet, in our hands, an overexpressed YopD-LcrH complex still strongly repressed *ysc-yop* T3SS in our Δ*pnp* mutant. These discrepancies probably reflect subtle genetic differences between the specific strains used in the various studies, which are impacted by the relative expression levels of the various RNases comprising the RNA degradosome. It also suggests that the role of PNPase in this regulatory process may not be a dominant feature in all *Yersinia* strains.

Another aspect of this study was the observation that YopD-LcrH complex can retain cytoplasmic pools of LcrQ. This is probably a consequence of the direct interaction between LcrQ and LcrH. This corroborates specific secretion of LcrQ occurring from regulatory-deficient mutants of *yopD* and *lcrH* when grown in the nonpermissive secretion conditions of plus Ca^2+^ (25, 48, 49). Interestingly, reciprocal experiments showed that YopD was specifically secreted in a Δ*lcrQ* strain grown in the same nonpermissive conditions (21, 29). This suggests that LcrQ may also retain critical cytoplasmic levels of YopD. The accumulation of cytoplasmic levels of both LcrQ and the YopD-LcrH complex would facilitate the repression of T3SS under noninducible conditions (Fig. 6). As LcrQ secretion is an obvious checkpoint in orchestrated control of Yop synthesis and secretion, an analysis of the LcrQ secretor domain is warranted. Precedent for the value of this type of study comes from an analysis of the equivalent YopD secretor domain that revealed features setting it aside from a classical T3SS substrate signal, including possible *yopD* translation control mechanisms (50).

Interestingly, we show that the negative regulatory function of YopD/LcrH was not completely abolished in the absence of LcrQ (Fig. 3D and E). However, the negative regulatory function of LcrQ was completely abolished in the absence of YopD-LcrH (Fig. 3B). This suggests that the regulatory role of LcrQ is strictly dependent on the presence of the YopD-LcrH complex, but the YopD-LcrH complex can function through both LcrQ-dependent and independent mechanisms. Our model of posttranscriptional regulation of *lcrF* expression reflects the involvement of these two pathways (Fig. 6). At this point, the reason for these two pathways and the relative contribution of each to regulatory control is not known. The LcrQ-independent nature of YopD function is thought to manifest itself in the form of translation inhibition of Yop synthesis by direct binding to *yop* mRNA (24), association with the 30S ribosomal subunit (27), and hijacking of global RNA regulators (26). However, these findings could be reinvestigated in light of LcrQ dependency.

Finally, we identified the LcrQ_L68A_ variant that had decreased ability to repress Yops synthesis and secretion despite maintaining an interaction with LcrH, RNase E, and RhlB. Although our interaction assay does not measure productive binding, we suggest that the phenotype associated with the LcrQ_L68A_ variant implies that LcrQ-dependent regulation must incorporate additional regulatory targets. In this context, we and others showed that LcrQ and/or YscM1/YscM2 can also directly interact with several other T3S chaperones, including SycH, SycE, SycO, and SycB (37, 51; this study). Furthermore, we demonstrated herein that LcrQ has potential to bind to itself. Despite the established importance of the LcrQ-SycH interaction to efficient LcrQ secretion (31, 33), roles for the other interactions in T3SS biogenesis, function, and regulation are not well established. However, all these interactions have potential to function in this regulatory process. Having access to the regulatory-deficient LcrQ_L68A_-producing mutant may provide an important genetic tool to revisit the biological consequences of these binding phenomena.

## MATERIALS AND METHODS

### Plasmids, bacterial strains, and growth conditions.

The Y. pseudotuberculosis YpIII and its derivate strains used in this study were cultured in YLB medium (1% tryptone, 0.5% NaCl, and 0.5% yeast extract) at 26°C. E. coli strains were grown in LB medium and incubated at 37°C for amplifying plasmids or at 20°C for protein expression. Ampicillin (100 μg/ml), kanamycin (50 μg/ml), and chloramphenicol (30 μg/ml) were supplemented to the medium when needed. All bacterial strains and plasmids used in this study are listed in Table S3 in the supplemental material.

10.1128/mBio.01457-21.7 TABLE S3 Strains and plasmids used in this study. Download Table S3, DOCX file, 0.04 MB. Copyright © 2021 Fei et al. 2021 Fei et al. https://creativecommons.org/licenses/by/4.0/ This content is distributed under the terms of the Creative Commons Attribution 4.0 International license.

### Plasmid construction.

All oligonucleotides used in this study are listed in Table S4. To construct the LcrQ overexpression plasmid, the *lcrQ* gene was cloned into the pOVR plasmid (34) between the PstI and KpnI sites to obtain the plasmid designated pOVR-LcrQ. A *gst*-encoding region was amplified and inserted upstream of the *lcrQ* gene in pOVR-LcrQ. To overexpress the YopD-LcrH complex, the *yopD* and *lcrH* genes were both amplified and overlapped into one fragment using a ribosomal binding region as an internal linker. This overlapped fragment was then cloned into the pOVR plasmid. Clones composed of various promoter-*lacZ* transcriptional fusions were constructed based on the pZT plasmid as described earlier (23). The promoter and 5′ UTR of *yopH* or *yopE* genes (35) were cloned upstream of promoterless *lacZ* using a ClonExpress II one step cloning kit (Vazyme). For the bacterial two-hybrid assay (52), genes were cloned into pKT25 or pUT18 using the ClonExpress II one step cloning kit (Vazyme).

10.1128/mBio.01457-21.8 TABLE S4 Primers used in this study. Download Table S4, DOCX file, 0.04 MB. Copyright © 2021 Fei et al. 2021 Fei et al. https://creativecommons.org/licenses/by/4.0/ This content is distributed under the terms of the Creative Commons Attribution 4.0 International license.

### Yops extraction and Western blotting assay.

The Yops produced by various YpIII strains were extracted as previously described (34, 53). Briefly, overnight cultures of YpIII strains in YLB were diluted (1:20) into Ca^2+^-depleted medium (20 mM MgCl_2_ and 5 mM EGTA) and cultured at 26°C for another 2 h. After that, cultures were transferred to 37°C and incubated for 4 h. Bacterial cell pellets were harvested by centrifugation. For each strain, an 8.1-ml supernatant fraction was carefully removed and then filtrated by a 0.22-μm filter to avoid bacterial contamination. Trichloroacetic acid (TCA) and acetone were used for protein precipitation from supernatant samples. The weights of bacterial cell pellets were determined for normalizing protein levels in bacterial pellets and supernatants. Proteins were dissolved in SDS-loading buffer and resolved by SDS-PAGE. For Western blotting, proteins resolved in SDS-PAGE were transferred into a polyvinylidene difluoride (PVDF) membrane (Millipore) by a semidry method. The membrane was then blocked with 5% nonfat milk. Protein-specific antiserum previously recovered from immunized rabbits (53) was diluted 1,000-fold and used to detect the protein levels of Yops. Mouse anti-Flag monoclonal antibody (1:2,000; Sigma) was used to detect the LcrF levels when it was fused with Flag tag. As appropriate, horseradish peroxidase (HRP)-labeled goat anti-rabbit or anti-mice IgG (1:10,000; Beyotime) was used as the secondary antibody. Enhanced chemiluminescence reagent (Bio-Rad) was used for signal generation. Image detection and collection used a ChemiDoc imaging system, and analysis was performed by the Image Lab software.

### Protein purification and GST pulldown assay.

E. coli strain BL21(DE3) was used for protein purification. The pET21a-LcrQ, pET21a-LcrQ3m, pGEX-KG, and pGEX-KG-LcrH plasmids were transformed into BL21(DE3) and the strains grown at 37°C in LB and incubated to an optical density of 0.4 at a wavelength of 600 nm. IPTG (isopropyl-β-d-thiogalactopyranoside) at a final concentration of 0.3 mM was used for protein production. Ni-NTA was used for His-LcrQ and His-LcrQ_F46A, L68A, L102A_ (His-LcrQ3m) purification, and glutathione Sepharose was used for GST and GST-LcrH purification. For the pulldown assay, His-LcrQ, GST-LcrH, His-LcrQ3m, and GST-LcrH were incubated at 37°C for 1 h. Ni-NTA was used to trap the complex via the His tag. The combinations of His-LcrQ and GST alone, as well as His-LcrQ3m and GST alone, were used as negative controls.

### YpIII mutant construction.

YpIII mutants or strains with integration of Flag tag at the 5′ end or 3′ end of the *lcrF* gene were constructed using the suicide plasmid pDM4 (54) as previously described (55). Briefly, an ∼500-bp fragment upstream and downstream of the region to be deleted was amplified, joined together by the two-step overlap PCR procedure, and then cloned into pDM4 plasmid. The pDM4 derivative was then transformed into E. coli S17‐1λ*pir* by chemical transformation and then conjugated into YpIII by conjugal mating. Allelic exchange by homologous recombination was screened as previously described (55).

### RNA isolation and qRT-PCR.

The culture conditions of strains were the same as used for Yops extraction. The TRIzol reagent (Ambion) was used for RNA isolation. The reverse transcription-quantitative PCR (qRT-PCR) assay was performed as described (56). Briefly, 2 μg DNase I (Promega)-treated RNA was used in reverse transcription assay with Moloney murine leukemia virus (M-MLV) reverse transcriptase (Promega). SYBR green supermix and CFX Connect fluorescence quantitative PCR detection system (Bio-Rad) were used in quantification assay. The copy number of 16S rRNA was used for normalization. For each gene expression analysis, at least three biological repetitions were performed, and each repetition contains two technical replicates.

### RNA stability assay.

The overnight cultures of YpIII strains in YLB were diluted (1:20) into fresh YLB with 20 mM MgCl_2_ and cultured at 26°C for 2 h, after which they were transferred to 37°C and incubated for a further 2 h. Rifampin was then added to a final concentration of 500 μg/ml. After determined time points (0 min, 2 min, 4 min, 6 min, and 8 min), samples were collected in the presence of 0.2 volumes of stop buffer (5% water-saturated phenol, 95% ethanol) and snap frozen in liquid nitrogen. RNA was isolated as described above, and the mRNA stability was detected by gene-specific qRT-PCR, also as described above.

### Bacteria two-hybrid assay.

The adenylate cyclase-based bacterial two-hybrid system was used to detect protein-protein interactions (52). E. coli BTH101 was cotransformed with various pKT25 and pUT18 derivatives. Three colonies from each transformation were used for testing the β-galactosidase activity using ONPG (*o*-nitrophenyl-β-d-galactopyranoside) (Songon) as the substrate. The empty plasmid pair of pKT25 and pUT18 was used as the negative control, and the pKT25-Zip and pUT18-Zip plasmid pair was used as the positive control. The β-galactosidase activity was examined according to previous descriptions (57).

### LcrQ mutant library screening.

For LcrQ point mutation library construction, the *lcrQ* gene was first translationally fused at the C terminus with mCherry and cloned into the pBAD22 plasmid (58). The site-directed point mutations of LcrQ were performed by following the protocol provided by QuikChange site-directed mutagenesis kit (Stratagene). All the altered amino acids were mutated to alanine (Ala). This mutant library was cotransformed with the pZT-*lcrG*p plasmid (34) into the Δ*lcrQ* mutant to test the repressive effect of the LcrQ protein. The β-galactosidase activity was monitored to indicate the *lcrG* promoter activity. The fluorescence intensity of mCherry (excitation and emission wavelengths are 587 nm and 610 nm, respectively) was measured by a microplate reader (Biotek) to indicate the expression level of the LcrQ variants. Three colonies were tested for each strain harboring a unique LcrQ variant. The relative fold repression of *lcrG*p by the LcrQ variants was calculated on the basis of *lcrG* promoter activity against the LcrQ expression level.

### Statistical analysis.

All data for the β-galactosidase activity assays were shown as mean ± standard deviation (SD) of the results of multiple independent experiments. Statistical analyses were performed using the unpaired Student's *t* test (two-tailed) between each of two groups.

## References

[1] Pha K, Navarro L. 2016. Yersinia type III effectors perturb host innate immune responses. World J Biol Chem 7:1–13. doi:10.4331/wjbc.v7.i1.1. 26981193 PMC4768113

[2] Grabowski B, Schmidt MA, Ruter C. 2017. Immunomodulatory Yersinia outer proteins (Yops)-useful tools for bacteria and humans alike. Virulence 8:1124–1147. doi:10.1080/21505594.2017.1303588. 28296562 PMC5711447

[3] Plano GV, Schesser K. 2013. The Yersinia pestis type III secretion system: expression, assembly and role in the evasion of host defenses. Immunol Res 57:237–245. doi:10.1007/s12026-013-8454-3. 24198067

[4] Cornelis GR, Boland A, Boyd AP, Geuijen C, Iriarte M, Neyt C, Sory MP, Stainier I. 1998. The virulence plasmid of Yersinia, an antihost genome. Microbiol Mol Biol Rev 62:1315–1352. doi:10.1128/MMBR.62.4.1315-1352.1998. 9841674 PMC98948

[5] Schesser Bartra S, Lorica C, Qian L, Gong X, Bahnan W, Barreras H, Jr., Hernandez R, Li Z, Plano GV, Schesser K. 2019. Chromosomally-encoded Yersinia pestis type III secretion effector proteins promote infection in cells and in mice. Front Cell Infect Microbiol 9:23. doi:10.3389/fcimb.2019.00023. 30854334 PMC6396649

[6] Deng W, Marshall NC, Rowland JL, McCoy JM, Worrall LJ, Santos AS, Strynadka NCJ, Finlay BB. 2017. Assembly, structure, function and regulation of type III secretion systems. Nat Rev Microbiol 15:323–337. doi:10.1038/nrmicro.2017.20. 28392566

[7] Dewoody RS, Merritt PM, Marketon MM. 2013. Regulation of the Yersinia type III secretion system: traffic control. Front Cell Infect Microbiol 3:4. doi:10.3389/fcimb.2013.00004. 23390616 PMC3565153

[8] Buttner D. 2012. Protein export according to schedule: architecture, assembly, and regulation of type III secretion systems from plant- and animal-pathogenic bacteria. Microbiol Mol Biol Rev 76:262–310. doi:10.1128/MMBR.05017-11. 22688814 PMC3372255

[9] Osborne SE, Coombes BK. 2011. Expression and secretion hierarchy in the nonflagellar type III secretion system. Future Microbiol 6:193–202. doi:10.2217/fmb.10.172. 21366419

[10] Hooker-Romero D, Mettert E, Schwiesow L, Balderas D, Alvarez PA, Kicin A, Gonzalez AL, Plano GV, Kiley PJ, Auerbuch V. 2019. Iron availability and oxygen tension regulate the Yersinia Ysc type III secretion system to enable disseminated infection. PLoS Pathog 15:e1008001. doi:10.1371/journal.ppat.1008001. 31869388 PMC6946166

[11] Miller HK, Kwuan L, Schwiesow L, Bernick DL, Mettert E, Ramirez HA, Ragle JM, Chan PP, Kiley PJ, Lowe TM, Auerbuch V. 2014. IscR is essential for yersinia pseudotuberculosis type III secretion and virulence. PLoS Pathog 10:e1004194. doi:10.1371/journal.ppat.1004194. 24945271 PMC4055776

[12] Fei K, Chao HJ, Hu Y, Francis MS, Chen S. 2021. CpxR regulates the Rcs phosphorelay system in controlling the Ysc-Yop type III secretion system in Yersinia pseudotuberculosis. Microbiology 167. doi:10.1099/mic.0.000998. 33295859

[13] Schiano CA, Lathem WW. 2012. Post-transcriptional regulation of gene expression in Yersinia species. Front Cell Infect Microbiol 2:129. doi:10.3389/fcimb.2012.00129. 23162797 PMC3493969

[14] Flores-Kim J, Darwin AJ. 2012. Links between type III secretion and extracytoplasmic stress responses in Yersinia. Front Cell Infect Microbiol 2:125. doi:10.3389/fcimb.2012.00125. 23087910 PMC3467454

[15] Knittel V, Vollmer I, Volk M, Dersch P. 2018. Discovering RNA-based regulatory systems for Yersinia virulence. Front Cell Infect Microbiol 8:378. doi:10.3389/fcimb.2018.00378. 30460205 PMC6232918

[16] Schwiesow L, Lam H, Dersch P, Auerbuch V. 2015. Yersinia type III secretion system master regulator LcrF. J Bacteriol 198:604–614. doi:10.1128/JB.00686-15. 26644429 PMC4751813

[17] Pettersson J, Nordfelth R, Dubinina E, Bergman T, Gustafsson M, Magnusson KE, Wolf-Watz H. 1996. Modulation of virulence factor expression by pathogen target cell contact. Science 273:1231–1233. doi:10.1126/science.273.5279.1231. 8703058

[18] Rosqvist R, Magnusson KE, Wolf-Watz H. 1994. Target cell contact triggers expression and polarized transfer of Yersinia YopE cytotoxin into mammalian-cells. EMBO J 13:964–972. doi:10.1002/j.1460-2075.1994.tb06341.x. 8112310 PMC394898

[19] Wattiau P, Cornelis GR. 1994. Identification of DNA-sequences recognized by Virf, the transcriptional activator of the Yersinia yop regulon. J Bacteriol 176:3878–3884. doi:10.1128/jb.176.13.3878-3884.1994. 8021169 PMC205584

[20] Hoe NP, Minion FC, Goguen JD. 1992. Temperature sensing in Yersinia pestis: regulation of yopE transcription by lcrF. J Bacteriol 174:4275–4286. doi:10.1128/jb.174.13.4275-4286.1992. 1624422 PMC206210

[21] Rimpiläinen M, Forsberg A, Wolf-Watz H. 1992. A novel protein, LcrQ, involved in the low-calcium response of Yersinia pseudotuberculosis shows extensive homology to YopH. J Bacteriol 174:3355–3363. doi:10.1128/jb.174.10.3355-3363.1992. 1577700 PMC206005

[22] Williams AW, Straley SC. 1998. YopD of Yersinia pestis plays a role in negative regulation of the low-calcium response in addition to its role in translocation of Yops. J Bacteriol 180:350–358. doi:10.1128/JB.180.2.350-358.1998. 9440524 PMC106890

[23] Anderson DM, Ramamurthi KS, Tam C, Schneewind O. 2002. YopD and LcrH regulate expression of Yersinia enterocolitica YopQ by a posttranscriptional mechanism and bind to yopQ RNA. J Bacteriol 184:1287–1295. doi:10.1128/JB.184.5.1287-1295.2002. 11844757 PMC134855

[24] Chen YQ, Anderson DM. 2011. Expression hierarchy in the Yersinia type III secretion system established through YopD recognition of RNA. Mol Microbiol 80:966–980. doi:10.1111/j.1365-2958.2011.07623.x. 21481017 PMC4128491

[25] Francis MS, Lloyd SA, Wolf-Watz H. 2001. The type III secretion chaperone LcrH co-operates with YopD to establish a negative, regulatory loop for control of Yop synthesis in Yersinia pseudotuberculosis. Mol Microbiol 42:1075–1093. doi:10.1046/j.1365-2958.2001.02702.x. 11737648

[26] Kusmierek M, Hoßmann J, Witte R, Opitz W, Vollmer I, Volk M, Heroven AK, Wolf-Watz H, Dersch P. 2019. A bacterial secreted translocator hijacks riboregulators to control type III secretion in response to host cell contact. PLoS Pathog 15:e1007813. doi:10.1371/journal.ppat.1007813. 31173606 PMC6583979

[27] Kopaskie KS, Ligtenberg KG, Schneewind O. 2013. Translational regulation of Yersinia enterocolitica mRNA encoding a type III secretion substrate. J Biol Chem 288:35478–35488. doi:10.1074/jbc.M113.504811. 24158443 PMC3853294

[28] Edqvist PJ, Broms JE, Betts HJ, Forsberg A, Pallen MJ, Francis MS. 2006. Tetratricopeptide repeats in the type III secretion chaperone, LcrH: their role in substrate binding and secretion. Mol Microbiol 59:31–44. doi:10.1111/j.1365-2958.2005.04923.x. 16359316

[29] Stainier I, Iriarte M, Cornelis GR. 1997. YscM1 and YscM2, two Yersinia enterocolitica proteins causing downregulation of yop transcription. Mol Microbiol 26:833–843. doi:10.1046/j.1365-2958.1997.6281995.x. 9427412

[30] Wulff-Strobel CR, Williams AW, Straley SC. 2002. LcrQ and SycH function together at the Ysc type III secretion system in Yersinia pestis to impose a hierarchy of secretion. Mol Microbiol 43:411–423. doi:10.1046/j.1365-2958.2002.02752.x. 11985718

[31] Cambronne ED, Cheng LW, Schneewind O. 2000. LcrQ/YscM1, regulators of the Yersinia yop virulon, are injected into host cells by a chaperone-dependent mechanism. Mol Microbiol 37:263–273. doi:10.1046/j.1365-2958.2000.01974.x. 10931323

[32] Wattiau P, Bernier B, Deslee P, Michiels T, Cornelis GR. 1994. Individual chaperones required for Yop secretion by Yersinia. Proc Natl Acad Sci U S A 91:10493–10497. doi:10.1073/pnas.91.22.10493. 7937981 PMC45047

[33] Cambronne ED, Sorg JA, Schneewind O. 2004. Binding of SycH chaperone to YscM1 and YscM2 activates effector yop expression in Yersinia enterocolitica. J Bacteriol 186:829–841. doi:10.1128/JB.186.3.829-841.2004. 14729710 PMC321491

[34] Li LM, Yan H, Feng LP, Li YL, Lu P, Hu YB, Chen SY. 2014. LcrQ blocks the role of LcrF in regulating the Ysc-Yop type III secretion genes in Yersinia pseudotuberculosis. PLoS One 9:e92243. doi:10.1371/journal.pone.0092243. 24658611 PMC3962397

[35] Cambronne ED, Schneewind O. 2002. Yersinia enterocolitica type III secretion: yscM1 and yscM2 regulate yop gene expression by a posttranscriptional mechanism that targets the 5' untranslated region of yop mRNA. J Bacteriol 184:5880–5893. doi:10.1128/JB.184.21.5880-5893.2002. 12374821 PMC135404

[36] Lu P, Zhang Y, Hu Y, Francis MS, Chen S. 2014. A cis-encoded sRNA controls the expression of fabH2 in Yersinia. FEBS Lett 588:1961–1966. doi:10.1016/j.febslet.2014.04.005. 24735725

[37] Swietnicki W, O'Brien S, Holman K, Cherry S, Brueggemann E, Tropea JE, Hines HB, Waugh DS, Ulrich RG. 2004. Novel protein-protein interactions of the Yersinia pestis type III secretion system elucidated with a matrix analysis by surface plasmon resonance and mass spectrometry. J Biol Chem 279:38693–38700. doi:10.1074/jbc.M405217200. 15213222

[38] Schmid A, Dittmann S, Grimminger V, Walter S, Heesemann J, Wilharm G. 2006. Yersinia enterocolitica type III secretion chaperone SycD: recombinant expression, purification and characterization of a homodimer. Protein Expr Purif 49:176–182. doi:10.1016/j.pep.2006.04.012. 16750393

[39] Bandyra KJ, Wandzik JM, Luisi BF. 2018. Substrate recognition and autoinhibition in the central ribonuclease RNase E. Mol Cell 72:275–285.e4. doi:10.1016/j.molcel.2018.08.039. 30270108 PMC6202311

[40] Chao Y, Li L, Girodat D, Förstner KU, Said N, Corcoran C, Śmiga M, Papenfort K, Reinhardt R, Wieden H-J, Luisi BF, Vogel J. 2017. In vivo cleavage map illuminates the central role of RNase E in coding and non-coding RNA pathways. Mol Cell 65:39–51. doi:10.1016/j.molcel.2016.11.002. 28061332 PMC5222698

[41] Yang J, Jain C, Schesser K. 2008. RNase E regulates the Yersinia type 3 secretion system. J Bacteriol 190:3774–3778. doi:10.1128/JB.00147-08. 18359811 PMC2395017

[42] Lodato PB, Thuraisamy T, Richards J, Belasco JG. 2017. Effect of RNase E deficiency on translocon protein synthesis in an RNase E-inducible strain of enterohemorrhagic Escherichia coli O157:H7. FEMS Microbiol Lett 364:fnx131. doi:10.1093/femsle/fnx131. PMC5827626 28854682

[43] Lodato PB, Hsieh PK, Belasco JG, Kaper JB. 2012. The ribosome binding site of a mini-ORF protects a T3SS mRNA from degradation by RNase E. Mol Microbiol 86:1167–1182. doi:10.1111/mmi.12050. 23043360 PMC3537511

[44] Sharp JS, Rietsch A, Dove SL. 2019. RNase E promotes expression of type III secretion system genes in Pseudomonas aeruginosa. J Bacteriol 201:e00336-19. doi:10.1128/JB.00336-19. 31481542 PMC6805110

[45] Bandyra KJ, Luisi BF. 2018. RNase E and the high-fidelity orchestration of RNA metabolism. Microbiol Spectr 6. doi:10.1128/microbiolspec.RWR-0008-2017. PMC11633573 29676248

[46] Rosenzweig JA, Chromy B, Echeverry A, Yang J, Adkins B, Plano GV, McCutchen-Maloney S, Schesser K. 2007. Polynucleotide phosphorylase independently controls virulence factor expression levels and export in Yersinia spp. FEMS Microbiol Lett 270:255–264. doi:10.1111/j.1574-6968.2007.00689.x. 17391372

[47] Rosenzweig JA, Weltman G, Plano GV, Schesser K. 2005. Modulation of yersinia type three secretion system by the S1 domain of polynucleotide phosphorylase. J Biol Chem 280:156–163. doi:10.1074/jbc.M405662200. 15509583

[48] Skrzypek E, Straley SC. 1995. Differential effects of deletions in lcrV on secretion of V antigen, regulation of the low-Ca2+ response, and virulence of Yersinia pestis. J Bacteriol 177:2530–2542. doi:10.1128/jb.177.9.2530-2542.1995. 7730287 PMC176914

[49] Broms JE, Francis MS, Forsberg A. 2007. Diminished LcrV secretion attenuates Yersinia pseudotuberculosis virulence. J Bacteriol 189:8417–8429. doi:10.1128/JB.00936-07. 17873031 PMC2168923

[50] Amer AA, Ahlund MK, Broms JE, Forsberg A, Francis MS. 2011. Impact of the N-terminal secretor domain on YopD translocator function in Yersinia pseudotuberculosis type III secretion. J Bacteriol 193:6683–6700. doi:10.1128/JB.00210-11. 21965570 PMC3232875

[51] Dittmann S, Schmid A, Richter S, Trulzsch K, Heesemann J, Wilharm G. 2007. The Yersinia enterocolitica type three secretion chaperone SycO is integrated into the Yop regulatory network and binds to the Yop secretion protein YscM1. BMC Microbiol 7:67. doi:10.1186/1471-2180-7-67. 17612396 PMC1933539

[52] Karimova G, Pidoux J, Ullmann A, Ladant D. 1998. A bacterial two-hybrid system based on a reconstituted signal transduction pathway. Proc Natl Acad Sci U S A 95:5752–5756. doi:10.1073/pnas.95.10.5752. 9576956 PMC20451

[53] Li Y, Li L, Huang L, Francis MS, Hu Y, Chen S. 2014. Yersinia Ysc-Yop type III secretion feedback inhibition is relieved through YscV-dependent recognition and secretion of LcrQ. Mol Microbiol 91:494–507. doi:10.1111/mmi.12474. 24344819

[54] Milton DL, O'Toole R, Horstedt P, Wolf-Watz H. 1996. Flagellin A is essential for the virulence of Vibrio anguillarum. J Bacteriol 178:1310–1319. doi:10.1128/jb.178.5.1310-1319.1996. 8631707 PMC177804

[55] O'Toole R, Milton DL, Wolf-Watz H. 1996. Chemotactic motility is required for invasion of the host by the fish pathogen Vibrio anguillarum. Mol Microbiol 19:625–637. doi:10.1046/j.1365-2958.1996.412927.x. 8830252

[56] Li YL, Hu YB, Francis MS, Chen SY. 2015. RcsB positively regulates the Yersinia Ysc-Yop type III secretion system by activating expression of the master transcriptional regulator LcrF. Environ Microbiol 17:1219–1233. doi:10.1111/1462-2920.12556. 25039908

[57] Hu Y, Lu P, Wang Y, Ding L, Atkinson S, Chen S. 2009. OmpR positively regulates urease expression to enhance acid survival of Yersinia pseudotuberculosis. Microbiology (Reading) 155:2522–2531. doi:10.1099/mic.0.028381-0. 19443542

[58] Guzman LM, Belin D, Carson MJ, Beckwith J. 1995. Tight regulation, modulation, and high-level expression by vectors containing the arabinose PBAD promoter. J Bacteriol 177:4121–4130. doi:10.1128/jb.177.14.4121-4130.1995. 7608087 PMC177145

