# Supplementary figures and images for: LcrQ Coordinates with the YopD-LcrH Complex To Repress lcrF Expression and Control Type III Secretion by Yersinia pseudotuberculosis
Source: mBio. 2021 Jun 22;12(3):e01457-21. doi: 10.1128/mBio.01457-21 (PMC8262909; doi:10.1128/mBio.01457-21)

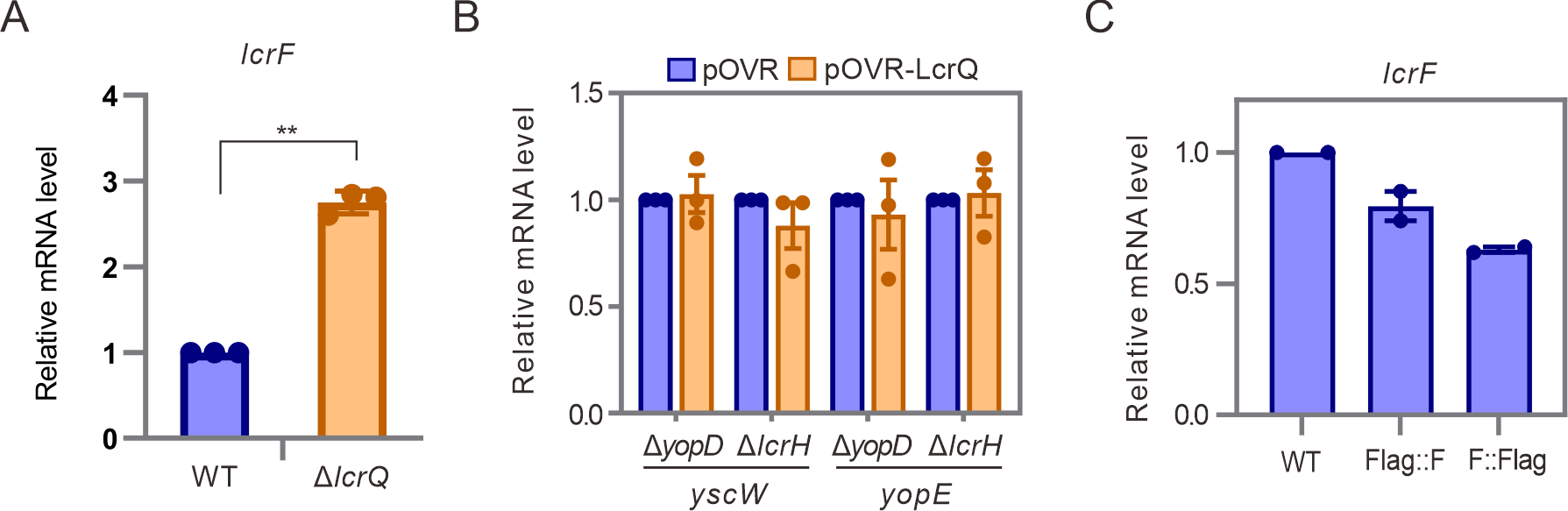

Supplement: FIG S1 [file mbio.01457-21-sf001.tif]

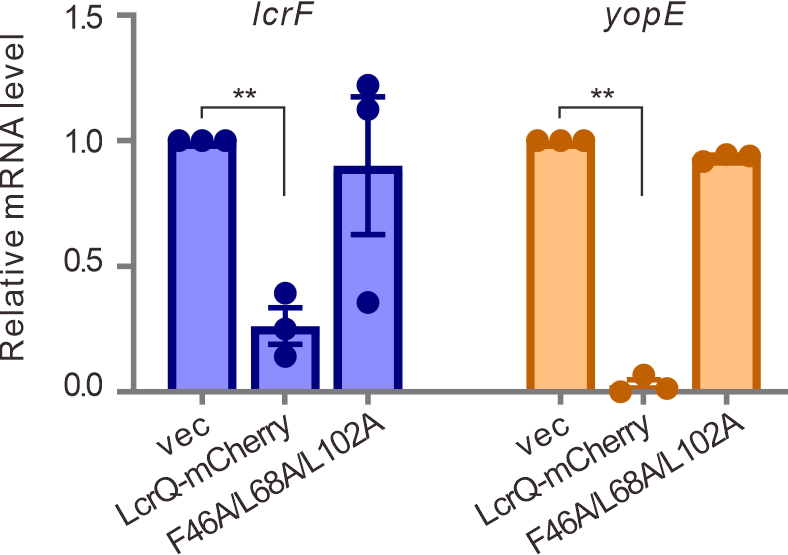

Supplement: FIG S2 [file mbio.01457-21-sf002.tif]

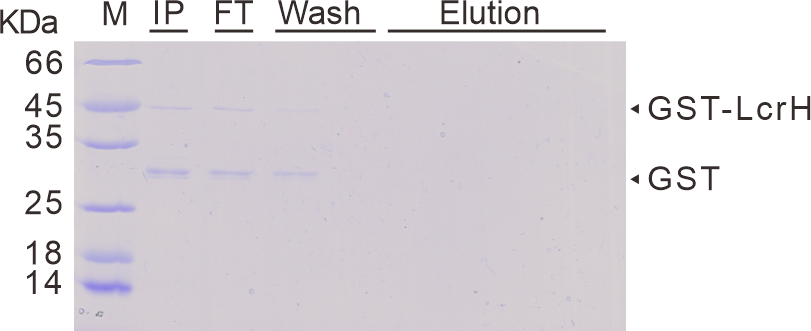

Supplement: FIG S3 [file mbio.01457-21-sf003.tif]

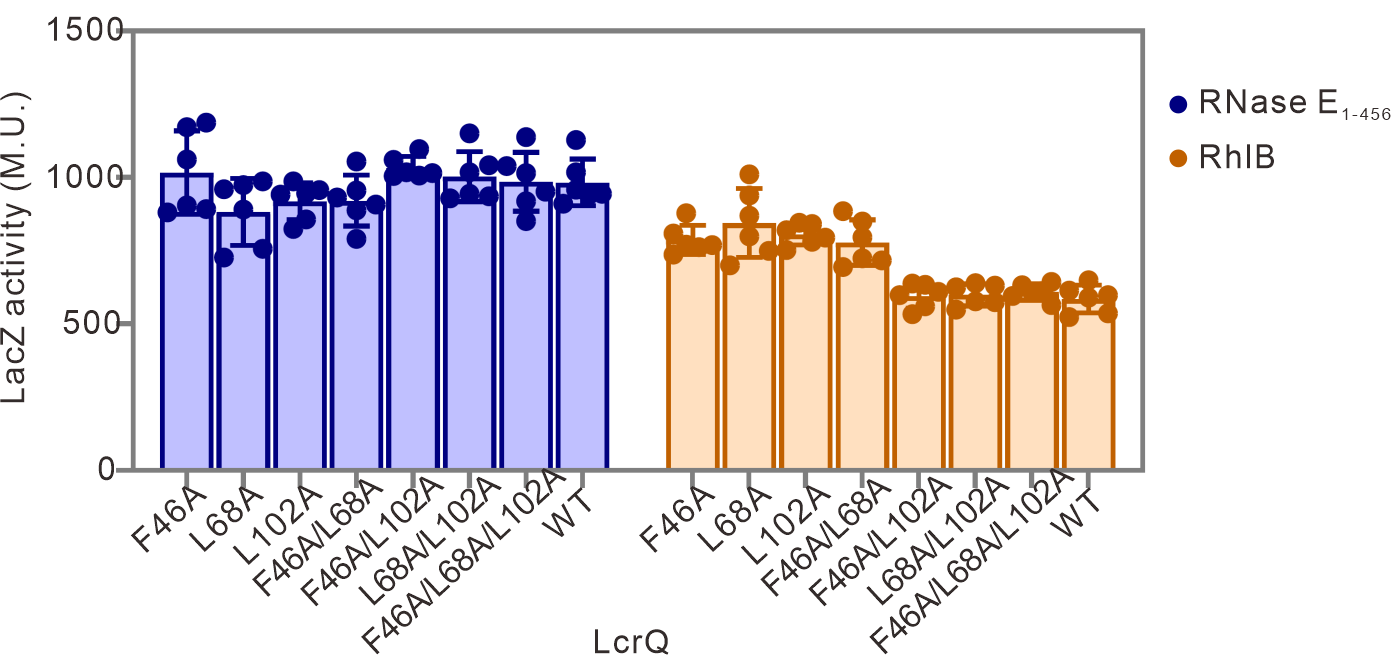

Supplement: FIG S4 [file mbio.01457-21-sf004.tif]
