# Supplementary material for: LcrQ Coordinates with the YopD-LcrH Complex To Repress lcrF Expression and Control Type III Secretion by Yersinia pseudotuberculosis
Source: mBio. 2021 Jun 22;12(3):e01457-21. doi: 10.1128/mBio.01457-21 (PMC8262909; doi:10.1128/mBio.01457-21)
Supplement: TABLE S1 [file mbio.01457-21-st001.docx]

**Table S1.** Expression levels of LcrQ single point mutants and their repression effects to the *lcrG* promoter activities.

|  | Enzyme activity | Repression fold | Fluorescence quantity | Relative expression level | Relative repression fold |
| --- | --- | --- | --- | --- | --- |
| vec | 30000.0 | 1.0 | 570.4 |  |  |
| WT | 953.3 | 31.5 | 11472.0 | 1.0 | 31.5 |
| K2A | 3600.0 | 8.3 | 6444.1 | 0.6 | 14.8 |
| I3A | 1370.6 | 21.9 | 2921.9 | 0.3 | 85.9 |
| N4A | 1009.0 | 29.7 | 10026.5 | 0.9 | 34.0 |
| T5A | 1192.8 | 25.2 | 7868.7 | 0.7 | 36.7 |
| L6A | 5425.2 | 5.5 | 1504.2 | 0.1 | 42.2 |
| Q7A | 3092.0 | 9.7 | 1056.9 | 0.1 | 105.3 |
| S8A | 12000.0 | 2.5 | 1505.4 | 0.1 | 19.1 |
| L9A | 1638.4 | 18.3 | 4553.1 | 0.4 | 46.1 |
| I10A | 8399.5 | 3.6 | 878.0 | 0.1 | 46.7 |
| N11A | 726.5 | 41.3 | 1427.1 | 0.1 | 332.0 |
| Q12A | 1232.1 | 24.3 | 2544.7 | 0.2 | 109.8 |
| Q13A | 1269.3 | 23.6 | 6870.6 | 0.6 | 39.5 |
| I14A | 1389.6 | 21.6 | 7624.7 | 0.7 | 32.5 |
| T15A | 1037.2 | 28.9 | 9822.5 | 0.9 | 33.8 |
| Q16A | 755.3 | 39.7 | 9091.8 | 0.8 | 50.1 |
| V17A | 1007.9 | 29.8 | 9759.5 | 0.9 | 35.0 |
| G18A | 1444.4 | 20.8 | 7935.8 | 0.7 | 30.0 |
| H19A | 1579.8 | 19.0 | 9153.2 | 0.8 | 23.8 |
| G20A | 1283.4 | 23.4 | 8861.6 | 0.8 | 30.3 |
| G21A | 1528.2 | 19.6 | 10185.2 | 0.9 | 22.1 |
| Q22A | 2034.2 | 14.7 | 7598.3 | 0.7 | 22.3 |
| G24A | 2231.0 | 13.4 | 7797.2 | 0.7 | 19.8 |
| R25A | 1354.5 | 22.1 | 10390.4 | 0.9 | 24.5 |
| L26A | 1268.0 | 23.7 | 8933.4 | 0.8 | 30.4 |
| T27A | 833.0 | 36.0 | 7685.4 | 0.7 | 53.8 |
| Q28A | 891.4 | 33.7 | 10661.2 | 0.9 | 36.2 |
| T29A | 1146.1 | 26.2 | 10436.9 | 0.9 | 28.8 |
| N30A | 1060.2 | 28.3 | 7422.0 | 0.6 | 43.7 |
| P31A | 1035.9 | 29.0 | 10372.8 | 0.9 | 32.0 |
| L32A | 1198.7 | 25.0 | 7733.8 | 0.7 | 37.1 |
| T33A | 1289.9 | 23.3 | 11349.7 | 1.0 | 23.5 |
| E34A | 1785.0 | 16.8 | 9743.8 | 0.8 | 19.8 |
| N35A | 684.1 | 43.9 | 7811.1 | 0.7 | 64.4 |
| S36A | 986.7 | 30.4 | 8019.6 | 0.7 | 43.5 |
| H37A | 1377.9 | 21.8 | 8700.3 | 0.8 | 28.7 |
| Q38A | 1446.3 | 20.7 | 5677.3 | 0.5 | 41.9 |
| I39A | 1087.8 | 27.6 | 10342.1 | 0.9 | 30.6 |
| S40A | 2278.2 | 13.2 | 9023.5 | 0.8 | 16.7 |
| T41A | 1015.0 | 29.6 | 10010.7 | 0.9 | 33.9 |
| E43A | 1276.4 | 23.5 | 10920.0 | 1.0 | 24.7 |
| K44A | 1038.3 | 28.9 | 13184.5 | 1.1 | 25.1 |
| F46A | 5111.9 | 5.9 | 14578.2 | 1.3 | 4.6 |
| N48A | 5865.0 | 5.1 | 5885.7 | 0.5 | 10.0 |
| E49A | 955.2 | 31.4 | 9886.3 | 0.9 | 36.4 |
| V50A | 3757.7 | 8.0 | 5944.0 | 0.5 | 15.4 |
| L51A | 890.0 | 33.7 | 6453.7 | 0.6 | 59.9 |
| E52A | 618.3 | 48.5 | 10376.3 | 0.9 | 53.6 |
| H53A | 1443.9 | 20.8 | 6457.7 | 0.6 | 36.9 |
| V54A | 18350.7 | 1.6 | 933.9 | 0.1 | 20.1 |
| K55A | 1684.2 | 17.8 | 9361.1 | 0.8 | 21.8 |
| N56A | 1253.8 | 23.9 | 6125.0 | 0.5 | 44.8 |
| T57A | 1200.6 | 25.0 | 10316.9 | 0.9 | 27.8 |
| L59A | 6134.4 | 4.9 | 6928.5 | 0.6 | 8.1 |
| S60A | 1410.6 | 21.3 | 15015.2 | 1.3 | 16.2 |
| R61A | 1777.6 | 16.9 | 11410.2 | 1.0 | 17.0 |
| H62A | 1587.2 | 18.9 | 10291.6 | 0.9 | 21.1 |
| D63A | 731.2 | 41.0 | 9300.8 | 0.8 | 50.6 |
| I64A | 2635.1 | 11.4 | 7740.6 | 0.7 | 16.9 |
| C66A | 830.4 | 36.1 | 10570.2 | 0.9 | 39.2 |
| L67A | 771.9 | 38.9 | 8113.5 | 0.7 | 55.0 |
| L68A | 6627.3 | 4.5 | 14551.5 | 1.3 | 3.6 |
| P69A | 647.6 | 46.3 | 9841.2 | 0.9 | 54.0 |
| R70A | 2163.9 | 13.9 | 13306.9 | 1.2 | 12.0 |
| V71A | 850.9 | 35.3 | 10700.3 | 0.9 | 37.8 |
| S72A | 1552.2 | 19.3 | 13049.1 | 1.1 | 17.0 |
| N73A | 928.0 | 32.3 | 7097.2 | 0.6 | 52.3 |
| L74A | 15692.6 | 1.9 | 1184.6 | 0.1 | 18.5 |
| E75A | 1371.8 | 21.9 | 11974.6 | 1.0 | 21.0 |
| L76A | 2769.8 | 10.8 | 14243.5 | 1.2 | 8.7 |
| K77A | 1773.9 | 16.9 | 14542.3 | 1.3 | 13.3 |
| Q78A | 1723.8 | 17.4 | 10297.2 | 0.9 | 19.4 |
| G79A | 2096.6 | 14.3 | 11550.1 | 1.0 | 14.2 |
| K80A | 1348.2 | 22.3 | 12615.6 | 1.1 | 20.2 |
| G82A | 981.8 | 30.6 | 9026.3 | 0.8 | 38.8 |
| E83A | 1293.3 | 23.2 | 8921.3 | 0.8 | 29.8 |
| V84A | 888.6 | 33.8 | 6612.3 | 0.6 | 58.6 |
| I85A | 2796.6 | 10.7 | 13512.1 | 1.2 | 9.1 |
| V86A | 3863.5 | 7.8 | 11913.9 | 1.0 | 7.5 |
| T87A | 1261.4 | 23.8 | 12900.2 | 1.1 | 21.1 |
| G88A | 2363.3 | 12.7 | 14294.1 | 1.2 | 10.2 |
| L89A | 1481.3 | 20.3 | 11682.7 | 1.0 | 19.9 |
| R90A | 2209.5 | 13.6 | 10867.2 | 0.9 | 14.3 |
| T91A | 1060.0 | 28.3 | 7388.1 | 0.6 | 43.9 |
| E92A | 962.9 | 31.2 | 10578.5 | 0.9 | 33.8 |
| Q93A | 2163.0 | 13.9 | 6899.7 | 0.6 | 23.1 |
| L94A | 1742.3 | 17.2 | 10128.4 | 0.9 | 19.5 |
| S95A | 1006.1 | 29.8 | 6942.6 | 0.6 | 49.3 |
| L96A | 1816.3 | 16.5 | 6321.8 | 0.6 | 30.0 |
| S97A | 972.8 | 30.8 | 14624.6 | 1.3 | 24.2 |
| D98A | 2994.5 | 10.0 | 12426.1 | 1.1 | 9.2 |
| K100A | 2782.7 | 10.8 | 6487.3 | 0.6 | 19.1 |
| L101A | 780.5 | 38.4 | 7625.0 | 0.7 | 57.8 |
| L102A | 5729.8 | 5.2 | 12947.1 | 1.1 | 4.6 |
| L103A | 2447.9 | 12.3 | 5570.8 | 0.5 | 25.2 |
| E104A | 2941.1 | 10.2 | 11777.8 | 1.0 | 9.9 |
| M107A | 967.3 | 31.0 | 12005.4 | 1.0 | 29.6 |
| R108A | 919.8 | 32.6 | 8173.7 | 0.7 | 45.8 |
| Q109A | 853.1 | 35.2 | 14045.8 | 1.2 | 28.7 |
| D110A | 1034.7 | 29.0 | 9920.1 | 0.9 | 33.5 |
| T111A | 1310.3 | 22.9 | 1230.4 | 0.1 | 213.5 |
| D114A | 1205.2 | 24.9 | 10444.1 | 0.9 | 27.3 |
| G115A | 1608.9 | 18.6 | 902.3 | 0.1 | 237.1 |
