# Supplementary material for: LcrQ Coordinates with the YopD-LcrH Complex To Repress lcrF Expression and Control Type III Secretion by Yersinia pseudotuberculosis
Source: mBio. 2021 Jun 22;12(3):e01457-21. doi: 10.1128/mBio.01457-21 (PMC8262909; doi:10.1128/mBio.01457-21)
Supplement: TABLE S2 [file mbio.01457-21-st002.docx]

**Table S2.** Expression levels of LcrQ double or triple mutants and their repression effects to the *lcrG* promoter activities.

|  | Enzyme activity | Repression fold | Fluorescence quantity | Relative expression level | Relative repression fold |
| --- | --- | --- | --- | --- | --- |
| vec | 32022.5 |  | 629.3 |  |  |
| WT | 382.0 | 83.8 | 16103.1 | 1.0 | 83.8 |
| F46A/L68A | 20797.1 | 1.5 | 11694.1 | 0.7 | 2.1 |
| F46A/L102A | 23641.4 | 1.4 | 16567.6 | 1.0 | 1.3 |
| L68A/L102A | 21907.2 | 1.5 | 13408.1 | 0.8 | 1.8 |
| F46A/L68A/L102A | 25756.1 | 1.2 | 16181.2 | 1.0 | 1.2 |
