# Supplementary material for: LcrQ Coordinates with the YopD-LcrH Complex To Repress lcrF Expression and Control Type III Secretion by Yersinia pseudotuberculosis
Source: mBio. 2021 Jun 22;12(3):e01457-21. doi: 10.1128/mBio.01457-21 (PMC8262909; doi:10.1128/mBio.01457-21)
Supplement: TABLE S3 [file mbio.01457-21-st003.docx]

**Table S3.** Strains and plasmids used in this study.

| **Bacteria** | **Relevant characteristics** | **Sources or references** |
| --- | --- | --- |
| ***Yersinia pseudotuberculosis*** | | |
| YpIII | *Y*. *pseudotuberculosis* YpIII (p+) strain | BEI Resources  NR-4380 |
| ∆*lcrQ* | Fragment of DNA encoding for amino acids 5-95was deleted | (53) |
| ∆*yopD* | Fragment of DNA encoding for amino acids was deleted | This study |
| ∆*lcrH* | Fragment of DNA encoding for amino acids was deleted | This study |
| ∆*yopH* | Fragment of DNA encoding for amino acids was deleted | This study |
| Δ*yopB* | Fragment of DNA encoding for amino acids was deleted | This study |
| Δ*sycH* | Fragment of DNA encoding for amino acids was deleted | This study |
| Δ*yopE* | Fragment of DNA encoding for amino acids was deleted | This study |
| Δ*sycE* | Fragment of DNA encoding for amino acids was deleted | This study |
| Δ*yopN* | Fragment of DNA encoding for amino acids was deleted | This study |
| Δ*yscB* | Fragment of DNA encoding for amino acids was deleted | This study |
| Δ*rnr* | Fragment of DNA encoding for amino acids was deleted | (36) |
| Δ*rne* | Fragment of DNA encoding for amino acids 701-1216 was deleted | (36) |
| Δ*rnb* | Fragment of DNA encoding for amino acids was deleted | (36) |
| Δ*pnp* | Fragment of DNA encoding for amino acids was deleted | (36) |
| *lcrF-Flag*:: *lcrF* | Flag tag is insert into the C-terminal of *lcrF* in genome | This study |
| *Flag-lcrF*::*lcrF* | Flag tag is insert into the N-terminal of *lcrF* in genome | This study |
| ***Escherichia coli*** | | |
| DH5α | *E. coli* strain used for cloning construction. | Lab collection |
| BL21(DE3) | *E. coli* strain used for protein expression. | Novagen |
| S17-1 | Sm^r^,Tp^r^, C600::RP-4 2-(Tc::Mu) (Kn::Tn7) *thi pro hsdR hsdM+ recA* | Lab collection |
| BTH101 | F^−^,*cya-99, ara*D139, *gal*E15, *gal*K16, *rps*L1^+^, *hsd*R2, *mcr*A1, *mcr*B1 | Lab collection |
| **Plasmids** | | |
| pGEX-KG-LcrH | pGEX-KG carrying *lcrH* gene from YPIII, Amp^R^ | This study |
| pET21a-LcrQ | Expressing *lcrQ* gene in pET-21a vector, Amp^R^ | This study |
| pET21a-LcrQ3m | Expressing *lcrQ3m* (F46A/L68A/L102A) gene in pET-21a vector, Amp^R^ | This study |
| pOVR | Expressing vector, pBR322 origin, *lac* promoter, Amp^R^ | (34) |
| pOVR-LcrQ | pOVR200 carrying *lcrQ* gene from YPIII, Amp^R^ | (34) |
| pOVR-YopD-LcrH | pOVR200 carrying *yopD* and *lcrH* gene from YPIII, Amp^R^ | This study |
| pOVR-GST-LcrQ | pOVR200 carrying *GST*-*lcrQ* fusion, Amp^R^ | (34) |
| pBAD22 | Expression vector, L-arabinose inducible, Amp^R^ | (58) |
| pBAD-LcrQ-mCherry | pBAD22 carrying *lcrQ-mCherry* fusion, Amp^R^ | This study |
| pBAD-LcrQ-mCherry library | pBAD-LcrQ-mCherry carrying mutations in LcrQ encoding region | This study |
| pDM4 | Suicide vector, mobRK2, oriR6K, sacBR of Bacillus subtilis, Cm^R^ | (54) |
| pDM4-LcrF-Flag | pDM4 carrying two DNA fragments up- and down-stream of position of Flag inserted in the3` of *lcrF*, Cm^R^ | This study |
| pDM4-Flag-LcrF | pDM4 carrying two DNA fragments up- and down-stream of position of Flag inserted in the 5`of *lcrF*, Cm^R^ | This study |
| pZT100 | Plasmid for promoter cloning, carrying a promoter-less lacZ as reporter, Kan^R^ | (34) |
| pZT-P*_yopE_*-UTR*_yopE_* | pZT100 carrying *yopE* promotor and 5`UTR, Kan^R^ | This study |
| pZT-P*_yopE_*-UTR*_lacZ_* | pZT100 carrying *yopE* promotor and *lacZ* 5`UTR, Kan^R^ | This study |
| pZT-P*_lacZ_*-UTR*_yopE_* | pZT100 carrying *lacZ* promotor and *yopE* 5`UTR, Kan^R^ | This study |
| pZT-P*_lacZ_*-UTR*_lacZ_* | pZT100 carrying *lacZ* promotor and *lacZ* 5`UTR, Kan^R^ | This study |
| pZT-P*_yopH_*-UTR*_yopH_* | pZT100 carrying *yopH* promotor and *yopH* 5`UTR, Kan^R^ | This study |
| pZT-P*_yopH_*-UTR*_lacZ_* | pZT100 carrying *yopH* promotor and *lacZ* 5`UTR, Kan^R^ | This study |
| pZT-P*_lacZ_*-UTR*_yopH_* | pZT100 carrying *lacZ* promotor and *yopH* 5`UTR, Kan^R^ | This study |
| pUT18 | Vector of bacterial two-hybrid system carrying T18 fragment of adenylate cyclase, Amp^R^ | (52) |
| pUT18-LcrQ | pBT18 carrying *lcrQ* gene，Kan^R^ | This study |
| pUT18-LcrH | pBT18 carrying *lcrH* gene，Kan^R^ | This study |
| pUT18-YopD | pBT18 carrying *yopD* gene，Kan^R^ | This study |
| pKT25 | Vector of bacterial two-hybrid system carrying T25 fragment of adenylate cyclase, Kan^R^ | (52) |
| pKT25-RNase_1-465_ | pKT25 carrying *rne* gene(1-1395 bp), Kan^R^ | This study |
| pKT25-RNase_400-1612_ | pKT25 carrying *rne* gene(1197-3651 bp), Kan^R^ | This study |
| pKT25-RhlB | pKT25 carrying *rhlB* gene, Kan^R^ | This study |
| pKT25-pYV library | pKT25 carrying *pYV0001 to pYV0099* genes, Kan^R^ | This study |
| pKT25-Zip | pKT25 carrying a fragment encoding Zip, Kan^R^ | (52) |
