# Supplementary material for: LcrQ Coordinates with the YopD-LcrH Complex To Repress lcrF Expression and Control Type III Secretion by Yersinia pseudotuberculosis
Source: mBio. 2021 Jun 22;12(3):e01457-21. doi: 10.1128/mBio.01457-21 (PMC8262909; doi:10.1128/mBio.01457-21)
Supplement: TABLE S4 [file mbio.01457-21-st004.docx]

**Table S4.** Primers used in this study.

| **Application** | **Name** | **Sequence (5'-3')** |
| --- | --- | --- |
| pZT cloning construction | pZT102-F | ATGACCATGATTACGGATTCACTG |
|  | pZT102-R | ATGTATATCTCCTTCTCTAGAGTCG |
|  | Placutr-F | CTAGAGAAGGAGATATACATCGATTCATTAATGCAGCTGGCACGA |
|  | pLacutr-R | CCAGTGAATCCGTAATCATGGTCATATGTATATCTCCTTCTTAAA |
|  | YopEputr-F | CTCTAGAGAAGGAGATATACATTTTGCCCGACAGGATGCTCTG |
|  | YopEputr-R | TGAATCCGTAATCATGGTCATGACTATTTATTACCTTGGCTATTA |
|  | YopEputr-xian-F | TTGTTTTAATAGCCAAGGTAATAAATA |
|  | pYopEputrlac-R | ATTACCTTGGCTATTAAAACAATCCACACAACATACGAGCCGGAAG |
|  | Placutr-xian-F | GAATTCAAATAATTTTGTTTAACTTTA |
|  | pYopEp-R | AGTTAAACAAAATTATTTGAATTCGGTTATCTTAGTGGGAAAATAGCCG |
|  | YopHputr-F | GACTCTAGAGAAGGAGATATACATATGCCGCCGTGGACGTTTCG |
|  | YopHputr-R | CCAGTGAATCCGTAATCATGGTCATGCTTCCCTCCTTAATTA |
|  | Lacutr-YopHp-R | GTTAAACAAAATTATTTGAATTCGCCTATACTATAAACATAAAAAAT |
|  | YopHutr-Lacp-R | CTTCCCTCCTTAATTAAATACACTCCACACAACATACGAGC |
| pOVR-YopD-LcrH | pOVR-F | GGTACCTTAAGCCAGCCCCG |
|  | pOVR-TSS-R | GTGTCTGAAAAACGTACCCTGATGGTGTACGTCCACACATTATACGAGCCGATG |
|  | pOVR-F2 | CGCAAGGAATGGTGCATGAAGC |
|  | pOVR-R2 | CGGGGCTGGCTTAAGGTACC |
| pET21a-LcrQ | PlcrQ-NF | GACAGCGGCATATGAAAATCAATACTCTTC |
|  | PlcrQ-XR | TTTCTCGAGGCCGTCAGCCGCCGTATC |
| pET21a-lcrQ3m | 21-Q-F | GAAGCCGCCATGCGCCAGGATACGGCGGCTG |
|  | 21-Q-RR | GTGAGTGGGTTAGTTTCAGTGAGACGACCG |
|  | LcrQ-F | CTCACTGAAACTAACCCACTCACAGAGAATAGTCATCAGATATCTACC |
|  | LcrQ-R | CGGCTTCTAGGGCTAATTTAGCATCGCTAAGCGAGAGTTGTTCAGTACG |
| pDM4-LcrF-Flag | pDM4-LcrF-Flag-F | AGCTCAGGTTACCCGCATGCAAGTCCACACCCAAACCATTGTTAATTT |
|  | pDM4-LcrF-Flag-R | CAGCGCTAGCGGAGTGTATATCAAGAACGGTTTTCTTCCTAAACTGGC |
|  | LcrF-Flag-D-F | CAAAGACGATGACGACAAGTAAAATTATCTGTGTTTTTTTTA |
|  | LcrF-Flag-U-R | TTACTTGTCGTCATCGTCTTTGTAGTCGCCTGTGGTTGCTATTTTAGTA |
| pDM4-Flag-LcrF | pDM4-Flag-LcrF-F | CAGCGCTAGCGGAGTGTATATCAAGGTGAGTCGTATTATAGCACTCATC |
|  | pDM4-Flag-LcrF-R | GAGCTCAGGTTACCCGCATGCAAGTCCTCCATAAATTTTTGCAACCGT |
|  | Flag-LcrF-D-F | CTACAAAGACGATGACGACAAGGCATCACTAGAGATTATTAAATTAG |
|  | Flag-LcrF-U-R | GTCGTCATCGTCTTTGTAGTCCATAAATGTTATACTGTCCTAAAAATC |
| pDM4-yscEm | PsycE-10up-SF | ATTGTCGACATTCAACTGCTGCATG |
|  | PsycE-10up-R | TTGAGTGATAGCTTGTTCAAATGAATA |
|  | PsycE-down-F | AGCTATCACTCAAGTAGATTTTTGGTTG |
|  | PsycE-down-SR | TATGCATGCAGTAGCCTGGGCGTAA |
| pDM4-yopBm | PyopB-28up-SF | CAGGTCGACGAACTATCGCCATGCT |
|  | PyopB-28up-R | GGCGGGCGCTGGTGTCTCGACGTAG |
|  | PyopB-97Dn-F | ACCAGCGCCCGCCTTTACGCTTGCTTCA |
|  | PyopB-97Dn-SR | TCCGCATGCCGCAAGACTTGCGGTG |
| pDM4-yopEm | PyopE-up-F | GCTAGCGGAGTGTATATCAAGGCTGAGTATATAGTGAGTTATTAT |
|  | PyopE-up-R | CTTGTTTTTATCCATATCACATCATGACTATTTATTACCTTGGCTATT |
|  | PyopE-dn-F | ATAGCCAAGGTAATAAATAGTCATGATGTGATATGGATAAAAACAAGGG |
|  | PyopE-dn-R | GAGCTCAGGTTACCCGCATGCAAGGCAGGAACAAATGTACCTGTGAG |
| pDM4-lcrHm | PlcrH-up-SF | AGCGTCGACGATTGACGTACTGTTA |
|  | PlcrH-up-R | AGAATGAGAAGATGGTGCATGAATT |
|  | PlcrH-dn-F | ATCTTCTCATTCTGAACTTGTAAATGCG |
|  | PlcrH-dn-SR | GAGATCTTGCAGTCCTTGCTAGAC |
| pDM4-yopDm | PyopD-U-SF | TAAGTCGACAGTCGCATTGACTGTA |
|  | PyopD-U-R | GGCTTCCTCCTTAAACTTAAACAG |
|  | PyopD-D-F | TTAAGGAGGAAGCCGTCTGACCATTGAT |
|  | PyopD-D-SR | GTTAGCATGCTATTTGGTTACGCCG |
| pDM4-sycHm | PsycH-U-SF | TAAGTCGACAGTCCCGACTGAAGCA |
|  | PsycH-U-R | ACTGTAAGTGCGCATATAACTGACC |
|  | PsycH-D-F | GCGCACTTACAGTTTCTATAAAAGAAAA |
|  | PsycH-D-SR | TTAGCATGCTGCTGGAGCATGGATT |
| pDM4-yopEm | PyscB-up-F | GCTAGCGGAGTGTATATCAAGCCAACCACAGGCTAAAATTATCTGTG |
|  | PyscB-up-R | CACGCGAGACGCTACAGAAAATGGTGTAACGTTATTTCCTTCACGTAACATTGGT |
|  | PyscB-dn-F | GTTACGTGAAGGAAATAACGTTACACCATTTTCTGTAGCGTCTCGCGTGGG |
|  | PyscB-dn-R | GAGCTCAGGTTACCCGCATGCAAGCAATCGCTAATGCCCCTGTTTTTTCACTG |
| pBAD-LcrQ-mCherry | pBAD-F | AAGCTTGGCTGTTTTGGCGG |
|  | pBAD-R | CATGGTGAATTCCTCCTGCTAG |
|  | LcrQ-Mcherry-F | GATACGGCGGCTGACGGCGGATCAGGATCAATGGTGAGCAAGGGCGAGGAG |
|  | LcrQ-Mcherry-R | CTCCTCGCCCTTGCTCACCATTGATCCTGATCCGCCGTCAGCCGCCGTATC |
|  | pBAD-LcrQ-F | GGCTAGCAGGAGGAATTCACCATGAAAATCAATACTCTTCAATCG |
|  | pBAD-Mcherry-R | CCGCCAAAACAGCCAAGCTTTTACTTGTACAGCTCGTCCATG |
| For LcrQ points mutant library | Qm-K2A-F | CTAGCAGGAGGAATTCACCATGGCCATCAATACTCTTCAATCG |
|  | Qm-K2A-R | TTAACGATTGAAGAGTATTGATGGCCATGGTGAATTCCTCCTG |
|  | Qm-I3A-F | GCAGGAGGAATTCACCATGAAAGCCAATACTCTTCAATCGTTA |
|  | Qm-I3A-R | TTATTAACGATTGAAGAGTATTGGCTTTCATGGTGAATTCCTC |
|  | Qm-N4A-F | GGAGGAATTCACCATGAAAATCGCCACTCTTCAATCGTTAATA |
|  | Qm-N4A-R | GATTTATTAACGATTGAAGAGTGGCGATTTTCATGGTGAATTC |
|  | Qm-T5A-F | GGAATTCACCATGAAAATCAATGCCCTTCAATCGTTAATAAAT |
|  | Qm-T5A-R | GTTGATTTATTAACGATTGAAGGGCATTGATTTTCATGGTGAA |
|  | Qm-L6A-F | ATTCACCATGAAAATCAATACTGCCCAATCGTTAATAAATCAA |
|  | Qm-L6A-R | TTTGTTGATTTATTAACGATTGGGCAGTATTGATTTTCATGGT |
|  | Qm-Q7A-F | CACCATGAAAATCAATACTCTTGCCTCGTTAATAAATCAACAA |
|  | Qm-Q7A-R | TAATTTGTTGATTTATTAACGAGGCAAGAGTATTGATTTTCAT |
|  | Qm-S8A-F | CATGAAAATCAATACTCTTCAAGCCTTAATAAATCAACAAATT |
|  | Qm-S8A-R | GGGTAATTTGTTGATTTATTAAGGCTTGAAGAGTATTGATTTT |
|  | Qm-L9A-F | GAAAATCAATACTCTTCAATCGGCCATAAATCAACAAATTACC |
|  | Qm-L9A-R | CTTGGGTAATTTGTTGATTTATGGCCGATTGAAGAGTATTGAT |
|  | Qm-I10A-F | AATCAATACTCTTCAATCGTTAGCCAATCAACAAATTACCCAA |
|  | Qm-I10A-R | CCACTTGGGTAATTTGTTGATTGGCTAACGATTGAAGAGTATT |
|  | Qm-N11A-F | CAATACTCTTCAATCGTTAATAGCCCAACAAATTACCCAAGTG |
|  | Qm-N11A-R | GTCCCACTTGGGTAATTTGTTGGGCTATTAACGATTGAAGAGT |
|  | Qm-Q12A-F | TACTCTTCAATCGTTAATAAATGCCCAAATTACCCAAGTGGGA |
|  | Qm-Q12A-R | CGTGTCCCACTTGGGTAATTTGGGCATTTATTAACGATTGAAG |
|  | Qm-Q13A-F | TCTTCAATCGTTAATAAATCAAGCCATTACCCAAGTGGGACAC |
|  | Qm-Q13A-R | CCCCGTGTCCCACTTGGGTAATGGCTTGATTTATTAACGATTG |
|  | Qm-I14A-F | TCAATCGTTAATAAATCAACAAGCCACCCAAGTGGGACACGGG |
|  | Qm-I14A-R | GCCCCCCGTGTCCCACTTGGGTGGCTTGTTGATTTATTAACGA |
|  | Qm-T15A-F | ATCGTTAATAAATCAACAAATTGCCCAAGTGGGACACGGGGGG |
|  | Qm-T15A-R | CCTGCCCCCCGTGTCCCACTTGGGCAATTTGTTGATTTATTAA |
|  | Qm-Q16A-F | GTTAATAAATCAACAAATTACCGCCGTGGGACACGGGGGGCAG |
|  | Qm-Q16A-R | CGGCCTGCCCCCCGTGTCCCACGGCGGTAATTTGTTGATTTAT |
|  | Qm-V17A-F | AATAAATCAACAAATTACCCAAGCCGGACACGGGGGGCAGGCC |
|  | Qm-V17A-R | GACCGGCCTGCCCCCCGTGTCCGGCTTGGGTAATTTGTTGATT |
|  | Qm-G18A-F | AAATCAACAAATTACCCAAGTGGCCCACGGGGGGCAGGCCGGT |
|  | Qm-G18A-R | GACGACCGGCCTGCCCCCCGTGGGCCACTTGGGTAATTTGTTG |
|  | Qm-H19A-F | TCAACAAATTACCCAAGTGGGAGCCGGGGGGCAGGCCGGTCGT |
|  | Qm-H19A-R | TGAGACGACCGGCCTGCCCCCCGGCTCCCACTTGGGTAATTTG |
|  | Qm-G20A-F | ACAAATTACCCAAGTGGGACACGCCGGGCAGGCCGGTCGTCTC |
|  | Qm-G20A-R | CAGTGAGACGACCGGCCTGCCCGGCGTGTCCCACTTGGGTAAT |
|  | Qm-G21A-F | AATTACCCAAGTGGGACACGGGGCCCAGGCCGGTCGTCTCACT |
|  | Qm-G21A-R | TTTCAGTGAGACGACCGGCCTGGGCCCCGTGTCCCACTTGGGT |
|  | Qm-Q22A-F | TACCCAAGTGGGACACGGGGGGGCCGCCGGTCGTCTCACTGAA |
|  | Qm-Q22A-R | TAGTTTCAGTGAGACGACCGGCGGCCCCCCCGTGTCCCACTTG |
|  | Qm-G24A-F | CCAAGTGGGACACGGGGGGCAGGCCGCCCGTCTCACTGAAACT |
|  | Qm-G24A-R | GGTTAGTTTCAGTGAGACGGGCGGCCTGCCCCCCGTGTCCCAC |
|  | Qm-R25A-F | AGTGGGACACGGGGGGCAGGCCGGTGCCCTCACTGAAACTAAC |
|  | Qm-R25A-R | GTGGGTTAGTTTCAGTGAGGGCACCGGCCTGCCCCCCGTGTCC |
|  | Qm-L26A-F | GGGACACGGGGGGCAGGCCGGTCGTGCCACTGAAACTAACCCA |
|  | Qm-L26A-R | TGAGTGGGTTAGTTTCAGTGGCACGACCGGCCTGCCCCCCGTG |
|  | Qm-T27A-F | ACACGGGGGGCAGGCCGGTCGTCTCGCCGAAACTAACCCACTC |
|  | Qm-T27A-R | CTGTGAGTGGGTTAGTTTCGGCGAGACGACCGGCCTGCCCCCC |
|  | Qm-Q28A-F | GGTCGTCTCACTGCCACTAACCCACTCACAGA |
|  | Qm-Q28A-R | TGAGTGGGTTAGTGGCAGTGAGACGACCGGCC |
|  | Qm-T29A-F | CGTCTCACTGAAGCCAACCCACTCACAGAGAA |
|  | Qm-T29A-R | CTGTGAGTGGGTTGGCTTCAGTGAGACGACCG |
|  | Qm-N30A-F | CTCACTGAAACTGCCCCACTCACAGAGAATAG |
|  | Qm-N30A-R | TCTCTGTGAGTGGGGCAGTTTCAGTGAGACGA |
|  | Qm-P31A-F | ACTGAAACTAACGCCCTCACAGAGAATAGTCA |
|  | Qm-P31A-R | TATTCTCTGTGAGGGCGTTAGTTTCAGTGAGA |
|  | Qm-L32A-F | GAAACTAACCCAGCCACAGAGAATAGTCATCA |
|  | Qm-L32A-R | GACTATTCTCTGTGGCTGGGTTAGTTTCAGTG |
|  | Qm-T33A-F | ACTAACCCACTCGCCGAGAATAGTCATCAGAT |
|  | Qm-T33A-R | GATGACTATTCTCGGCGAGTGGGTTAGTTTCA |
|  | Qm-E34A-F | AACCCACTCACAGCCAATAGTCATCAGATATC |
|  | Qm-E34A-R | TCTGATGACTATTGGCTGTGAGTGGGTTAGTT |
|  | Qm-N35A-F | CCACTCACAGAGGCCAGTCATCAGATATCTAC |
|  | Qm-N35A-R | ATATCTGATGACTGGCCTCTGTGAGTGGGTTA |
|  | Qm-S36A-F | CTCACAGAGAATGCCCATCAGATATCTACCGC |
|  | Qm-S36A-R | TAGATATCTGATGGGCATTCTCTGTGAGTGGG |
|  | Qm-H37A-F | ACAGAGAATAGTGCCCAGATATCTACCGCCGA |
|  | Qm-H37A-R | CGGTAGATATCTGGGCACTATTCTCTGTGAGT |
|  | Qm-Q38A-F | GAGAATAGTCATGCCATATCTACCGCCGAAAAAGC |
|  | Qm-Q38A-R | CGGCGGTAGATATGGCATGACTATTCTCTGTG |
|  | Qm-I39A-F | GAATAGTCATCAGGCCTCTACCGCCGAAAAAGC |
|  | Qm-I39A-R | TTTCGGCGGTAGAGGCCTGATGACTATTCTCT |
|  | Qm-S40A-F | TAGTCATCAGATAGCCACCGCCGAAAAAGCCT |
|  | Qm-S40A-R | CTTTTTCGGCGGTGGCTATCTGATGACTATTC |
|  | Qm-T41A-F | TCATCAGATATCTGCCGCCGAAAAAGCCTTTG |
|  | Qm-T41A-R | AGGCTTTTTCGGCGGCAGATATCTGATGACTA |
|  | Qm-E43A-F | GATATCTACCGCCGCCAAAGCCTTTGCCAATG |
|  | Qm-E43A-R | TGGCAAAGGCTTTGGCGGCGGTAGATATCTGA |
|  | Qm-K44A-F | ATCTACCGCCGAAGCCGCCTTTGCCAATGAGG |
|  | Qm-K44A-R | CATTGGCAAAGGCGGCTTCGGCGGTAGATATC |
|  | Qm-F46A-F | CGCCGAAAAAGCCGCCGCCAATGAGGTGCTGG |
|  | Qm-F46A-R | GCACCTCATTGGCGGCGGCTTTTTCGGCGGTA |
|  | Qm-N48A-F | AAAAGCCTTTGCCGCCGAGGTGCTGGAACATG |
|  | Qm-N48A-R | GTTCCAGCACCTCGGCGGCAAAGGCTTTTTCG |
|  | Qm-E49A-F | AGCCTTTGCCAATGCCGTGCTGGAACATGTGA |
|  | Qm-E49A-R | CATGTTCCAGCACGGCATTGGCAAAGGCTTTT |
|  | Qm-V50A-F | CTTTGCCAATGAGGCCCTGGAACATGTGAAAA |
|  | Qm-V50A-R | TCACATGTTCCAGGGCCTCATTGGCAAAGGCT |
|  | Qm-L51A-F | TGCCAATGAGGTGGCCGAACATGTGAAAAATA |
|  | Qm-L51A-R | TTTTCACATGTTCGGCCACCTCATTGGCAAAG |
|  | Qm-E52A-F | CAATGAGGTGCTGGCCCATGTGAAAAATACGG |
|  | Qm-E52A-R | TATTTTTCACATGGGCCAGCACCTCATTGGCA |
|  | Qm-H53A-F | TGAGGTGCTGGAAGCCGTGAAAAATACGGCTC |
|  | Qm-H53A-R | CCGTATTTTTCACGGCTTCCAGCACCTCATTG |
|  | Qm-V54A-F | GGTGCTGGAACATGCCAAAAATACGGCTCTCA |
|  | Qm-V54A-R | GAGCCGTATTTTTGGCATGTTCCAGCACCTCA |
|  | Qm-K55A-F | GCTGGAACATGTGGCCAATACGGCTCTCAGTC |
|  | Qm-K55A-R | TGAGAGCCGTATTGGCCACATGTTCCAGCACC |
|  | Qm-N56A-F | GGAACATGTGAAAGCCACGGCTCTCAGTCGTC |
|  | Qm-N56A-R | GACTGAGAGCCGTGGCTTTCACATGTTCCAGC |
|  | Qm-T57A-F | ACATGTGAAAAATGCCGCTCTCAGTCGTCACG |
|  | Qm-T57A-R | GACGACTGAGAGCGGCATTTTTCACATGTTCC |
|  | Qm-L59A-F | GAAAAATACGGCTGCCAGTCGTCACGATATTG |
|  | Qm-L59A-R | TATCGTGACGACTGGCAGCCGTATTTTTCACA |
|  | Qm-S60A-F | AAATACGGCTCTCGCCCGTCACGATATTGCCT |
|  | Qm-S60A-R | CAATATCGTGACGGGCGAGAGCCGTATTTTTC |
|  | Qm-R61A-F | GGCTCTCAGTGCCCACGATATTGCCTGCTTATTACC |
|  | Qm-R61A-R | GCAATATCGTGGGCACTGAGAGCCGTATTTTTCACATG |
|  | Qm-H62A-F | CTCTCAGTCGTGCCGATATTGCCTGCTTATTAC |
|  | Qm-H62A-R | CAGGCAATATCGGCACGACTGAGAGCCGTATTTTTC |
|  | Qm-D63A-F | CTCAGTCGTCACGCCATTGCCTGCTTATTAC |
|  | Qm-D63A-R | AGCAGGCAATGGCGTGACGACTGAGAGCCG |
|  | Qm-I64A-F | GTCGTCACGATGCCGCCTGCTTATTACCAC |
|  | Qm-I64A-R | ATAAGCAGGCGGCATCGTGACGACTGAGAG |
|  | Qm-C66A-F | CGATATTGCCGCCTTATTACCACGCGTTTC |
|  | Qm-C66A-R | GTGGTAATAAGGCGGCAATATCGTGACGAC |
|  | Qm-L67A-F | CACGATATTGCCTGCGCCTTACCACGCGTTTC |
|  | Qm-L67A-R | CGCGTGGTAAGGCGCAGGCAATATCGTGAC |
|  | Qm-L68A-F | TGCCTGCTTAGCCCCACGCGTTTCTAATTTG |
|  | Qm-L68A-R | GAAACGCGTGGGGCTAAGCAGGCAATATCGTG |
|  | Qm-P69A-F | GCCTGCTTATTAGCCCGCGTTTCTAATTTG |
|  | Qm-P69A-R | ATTAGAAACGCGGGCTAATAAGCAGGCAAT |
|  | Qm-R70A-F | TGCTTATTACCAGCCGTTTCTAATTTGGAA |
|  | Qm-R70A-R | CAAATTAGAAACGGCTGGTAATAAGCAGGC |
|  | Qm-V71A-F | TTATTACCACGCGCCTCTAATTTGGAACTA |
|  | Qm-V71A-R | TTCCAAATTAGAGGCGCGTGGTAATAAGCA |
|  | Qm-S72A-F | TTACCACGCGTTGCCAATTTGGAACTAAAG |
|  | Qm-S72A-R | TAGTTCCAAATTGGCAACGCGTGGTAATAA |
|  | Qm-N73A-F | CCACGCGTTTCTGCCTTGGAACTAAAGCAG |
|  | Qm-N73A-R | CTTTAGTTCCAAGGCAGAAACGCGTGGTAA |
|  | Qm-L74A-F | CGCGTTTCTAATGCCGAACTAAAGCAGGGC |
|  | Qm-L74A-R | CTGCTTTAGTTCGGCATTAGAAACGCGTGG |
|  | Qm-E75A-F | GTTTCTAATTTGGCCCTAAAGCAGGGCAAG |
|  | Qm-E75A-R | GCCCTGCTTTAGGGCCAAATTAGAAACGCG |
|  | Qm-L76A-F | TCTAATTTGGAAGCCAAGCAGGGCAAGGCA |
|  | Qm-L76A-R | CTTGCCCTGCTTGGCTTCCAAATTAGAAAC |
|  | Qm-K77A-F | AATTTGGAACTAGCCCAGGGCAAGGCAGGG |
|  | Qm-K77A-R | TGCCTTGCCCTGGGCTAGTTCCAAATTAGA |
|  | Qm-Q78A-F | TTGGAACTAAAGGCCGGCAAGGCAGGGGAA |
|  | Qm-Q78A-R | CCCTGCCTTGCCGGCCTTTAGTTCCAAATT |
|  | Qm-G79A-F | GAACTAAAGCAGGCCAAGGCAGGGGAAGTG |
|  | Qm-G79A-R | TTCCCCTGCCTTGGCCTGCTTTAGTTCCAA |
|  | Qm-K80A-F | CTAAAGCAGGGCGCCGCAGGGGAAGTGATA |
|  | Qm-K80A-R | CACTTCCCCTGCGGCGCCCTGCTTTAGTTC |
|  | Qm-G82A-F | CAGGGCAAGGCAGCCGAAGTGATAGTGACC |
|  | Qm-G82A-R | CACTATCACTTCGGCTGCCTTGCCCTGCTT |
|  | Qm-E83A-F | GGCAAGGCAGGGGCCGTGATAGTGACCGGC |
|  | Qm-E83A-R | GGTCACTATCACGGCCCCTGCCTTGCCCTG |
|  | Qm-V84A-F | AAGGCAGGGGAAGCCATAGTGACCGGCTTG |
|  | Qm-V84A-R | GCCGGTCACTATGGCTTCCCCTGCCTTGCC |
|  | Qm-I85A-F | GCAGGGGAAGTGGCCGTGACCGGCTTGCGT |
|  | Qm-I85A-R | CAAGCCGGTCACGGCCACTTCCCCTGCCTT |
|  | Qm-V86A-F | GGGGAAGTGATAGCCACCGGCTTGCGTACT |
|  | Qm-V86A-R | ACGCAAGCCGGTGGCTATCACTTCCCCTGC |
|  | Qm-T87A-F | GAAGTGATAGTGGCCGGCTTGCGTACTGAA |
|  | Qm-T87A-R | AGTACGCAAGCCGGCCACTATCACTTCCCC |
|  | Qm-G88A-F | GTGATAGTGACCGCCTTGCGTACTGAACAA |
|  | Qm-G88A-R | TTCAGTACGCAAGGCGGTCACTATCACTTC |
|  | Qm-L89A-F | ATAGTGACCGGCGCCCGTACTGAACAACTC |
|  | Qm-L89A-R | TTGTTCAGTACGGGCGCCGGTCACTATCAC |
|  | Qm-R90A-F | GTGACCGGCTTGGCCACTGAACAACTCTCG |
|  | Qm-R90A-R | GAGTTGTTCAGTGGCCAAGCCGGTCACTAT |
|  | Qm-T91A-F | ACCGGCTTGCGTGCCGAACAACTCTCGCTT |
|  | Qm-T91A-R | CGAGAGTTGTTCGGCACGCAAGCCGGTCAC |
|  | Qm-E92A-F | GGCTTGCGTACTGCCCAACTCTCGCTTAGC |
|  | Qm-E92A-R | AAGCGAGAGTTGGGCAGTACGCAAGCCGGT |
|  | Qm-Q93A-F | TTGCGTACTGAAGCCCTCTCGCTTAGCGAT |
|  | Qm-Q93A-R | GCTAAGCGAGAGGGCTTCAGTACGCAAGCC |
|  | Qm-L94A-F | CGTACTGAACAAGCCTCGCTTAGCGATGCT |
|  | Qm-L94A-R | ATCGCTAAGCGAGGCTTGTTCAGTACGCAA |
|  | Qm-S95A-F | ACTGAACAACTCGCCCTTAGCGATGCTAAA |
|  | Qm-S95A-R | AGCATCGCTAAGGGCGAGTTGTTCAGTACG |
|  | Qm-L96A-F | GAACAACTCTCGGCCAGCGATGCTAAATTA |
|  | Qm-L96A-R | TTTAGCATCGCTGGCCGAGAGTTGTTCAGT |
|  | Qm-S97A-F | CAACTCTCGCTTGCCGATGCTAAATTATTG |
|  | Qm-S97A-R | TAATTTAGCATCGGCAAGCGAGAGTTGTTC |
|  | Qm-D98A-F | CTCTCGCTTAGCGCCGCTAAATTATTGCTA |
|  | Qm-D98A-R | CAATAATTTAGCGGCGCTAAGCGAGAGTTG |
|  | Qm-K100A-F | CTTAGCGATGCTGCCTTATTGCTAGAAGCC |
|  | Qm-K100A-R | TTCTAGCAATAAGGCAGCATCGCTAAGCGA |
|  | Qm-L101A-F | AGCGATGCTAAAGCCTTGCTAGAAGCCGCC |
|  | Qm-L101A-R | GGCTTCTAGCAAGGCTTTAGCATCGCTAAG |
|  | Qm-L102A-F | GATGCTAAATTAGCCCTAGAAGCCGCCATG |
|  | Qm-L102A-R | GGCGGCTTCTAGGGCTAATTTAGCATCGCT |
|  | Qm-L103A-F | GCTAAATTATTGGCCGAAGCCGCCATGCGC |
|  | Qm-L103A-R | CATGGCGGCTTCGGCCAATAATTTAGCATC |
|  | Qm-E104A-F | AAATTATTGCTAGCCGCCGCCATGCGCCAG |
|  | Qm-E104A-R | GCGCATGGCGGCGGCTAGCAATAATTTAGC |
|  | Qm-M107A-F | CTAGAAGCCGCCGCCCGCCAGGATACGGCG |
|  | Qm-M107A-R | CGTATCCTGGCGGGCGGCGGCTTCTAGCAA |
|  | Qm-R108A-F | GAAGCCGCCATGGCCCAGGATACGGCGGCT |
|  | Qm-R108A-R | CGCCGTATCCTGGGCCATGGCGGCTTCTAG |
|  | Qm-Q109A-F | GCCGCCATGCGCGCCGATACGGCGGCTGAC |
|  | Qm-Q109A-R | AGCCGCCGTATCGGCGCGCATGGCGGCTTC |
|  | Qm-D110A-F | GCCATGCGCCAGGCCACGGCGGCTGACGGC |
|  | Qm-D110A-R | GTCAGCCGCCGTGGCCTGGCGCATGGCGGC |
|  | Qm-T111A-F | ATGCGCCAGGATGCCGCGGCTGACGGCGGA |
|  | Qm-T111A-R | GCCGTCAGCCGCGGCATCCTGGCGCATGGC |
|  | Qm-D114A-F | GATACGGCGGCTGCCGGCGGATCAGGATCA |
|  | Qm-D114A-R | TCCTGATCCGCCGGCAGCCGCCGTATCCTG |
|  | Qm-G115A-F | ACGGCGGCTGACGCCGGATCAGGATCAATG |
| For qRT-PCR | q16s-F | CCCCTGGACAAAGACTGA |
|  | q16s-R | AAGGGCACAACCTCCAAG |
|  | qVirG-R | AGCCACCGCCTGAACTCC |
|  | qVirG-F | CCATTATCTCGCATAGCACACA |
|  | qlcrF-F | TGCCAATGAGGTGCTGGA |
|  | qlcrF-R | CTGCCTTGCCCTGCTTTA |
|  | qYopD-F | TCTCAAGCCGAGGTCAAAGA |
|  | qYopD-R | TGTTCAATCAAGCGCAGGAC |
|  | qYopE-F | CGCATGTTCTCGGAGGG |
|  | qYopE-R | TTGGCAGCGTCTCAGCA |
|  | qYopH-F | GGTCTCGGTGACGGGATTA |
|  | qYopH-R | TGAAGCGAGTGCCTTGGTA |
|  | qpYV0023-F | GCAGAAGGCGTCGGAAAG |
|  | qpYV0023-R | GCAATGCTGTCCAGAATACGC |
|  | qLcrQ-F | TGCCAATGAGGTGCTGGA |
|  | qLcrQ-R | CTGCCTTGCCCTGCTTTA |
|  | qLcrF-F | GGGTCACACCTATATTTAAGGTTG |
|  | qLcrF-F | CCGGGTAATGGAATCCAAAG |
| pKT-pYV library | P001-SF | TAGGCATGCATGAAAAGCGTGAAAA |
|  | P001-SR | ATAGGTACCCACATCCATTCCCGCT |
|  | P002-PF | GCGCTGCAGATGATTAACACCACCT |
|  | P002-KR | TAAGGTACCCATCCCCATTTAACCG |
|  | P004-PF | GCCCTGCAGATGTCTAAAAAACACT |
|  | P004-BR | AAAGGATCCCAACATAGGGATAAGG |
|  | P006-PF | ATACTGCAGATGGGCCCCGGTAACC |
|  | P006-KR | GGCGGTACCCAGATTACCGATTGTT |
|  | P010-PF | ATACTGCAGATGGGCGGCATGGCCG |
|  | P010-KR | ACAGGTACCCACGTAAAGAGATGCA |
|  | P012-PF | TATCTGCAGATGGGGAACTACATGG |
|  | P012-KR | TAAGGTACCCAATCACACGGCTGAAA |
|  | P013-SF | GCCACTAGTATGACTAAAGATTTTAAGA |
|  | P013-KR | AACGGTACCCACCACTCGATATTAAAT |
|  | P020-PF | AATCTGCAGATGCGCACTTACAGTT |
|  | P020-KR | TTGGGTACCCAAACCAGTAAATGAG |
|  | P024-PF | CGCCTGCAGATGTATTCATTTGAAC |
|  | P024-KR | AATGGTACCCAACTAAATGACCGTG |
|  | P025-SF | GCGACTAGTATGAAAATATCATCAT |
|  | P025-KR | ATAGGTACCCACATCAATGACAGTA |
|  | P029-PF | TATCTGCAGATGAAATCGCCGTATC |
|  | P029-KR | TATGGTACCCAGCCCAGAGCTATTG |
|  | P030-PF | TATCTGCAGATGGATATCCAGGTGG |
|  | P030-KR | AGAGGTACCCACATTTCCACTAGGA |
|  | P033-PF | TTACTGCAGATGCCCAAGAGCCTGA |
|  | P033-KR | ATAGGTACCCAAACAGTCCCCAGAG |
|  | P036-PF | TTTCTGCAGATGAGCCGGGTCGTTA |
|  | P036-KR | ATCGGTACCCAACGCAAAGATTTGA |
|  | P040-PF | GGGCTGCAGATGTTTATTAAAGATAC |
|  | P040-KR | TAAGGTACCCATCCCATAATACATTC |
|  | P045-PF | ATACTGCAGATGGTAAACGCGGC |
|  | P045-KR | ATCGGTACCCAGGCTAAAACAACCT |
|  | P047-PF | GCTCTGCAGATGTATGGTTTTGTTT |
|  | P047-KR | GATGGTACCCACTCAAAAACATCAT |
|  | P048-PF | AAACTGCAGATGGCCGGTATTGAAACT |
|  | P048-KR | TCGGGTACCCAGAAGTATTGTATGG |
|  | P049-PF | TCACTGCAGATGAATAGCCTCAGTT |
|  | P049-KR | TATGGTACCCAATGTGAGTCTGCTT |
|  | P050-PF | TTTCTGCAGATGGCCCCAGGTGATT |
|  | P050-KR | CGCGGTACCCAATAAACTAACGGTA |
|  | P054-PF | GCCCTGCAGATGACAATAAATATCA |
|  | P054-KR | ATAGGTACCCAGACAACACCAAAAG |
|  | P055-PF | TTTCTGCAGATGAGTGCGTTGATAA |
|  | P055-KR | ATTGGTACCCAAACAGTATGGGGTC |
|  | P056-PF | AAACTGCAGATGCAACAAGAGACGA |
|  | P056-KR | AAAGGTACCCATGGGTTATCAACGC |
|  | P057-SF | GCCACTAGTATGATTAGAGCCTACGA |
|  | P057-KR | ATAGGTACCCATTTACCAGACGTGT |
|  | P058-PF | TCACTGCAGATGAAATCTTCCCATT |
|  | P058-KR | TATGGTACCCAAATAATTTGCCCTC |
|  | P059-SF | GGGACTAGTATGATGGCAGATCCTT |
|  | P059-KR | ATAGGTACCCAGGCAAAGCCGTTGT |
|  | P060-PF | TTACTGCAGATGAATCCCCATGATC |
|  | P060-XR | GGCTCTAGACATAAGCAAATTCGTCC |
|  | P061-PF | GCCCTGCAGATGAATATTACTTTAACC |
|  | P061-KR | TTAGGTACCCATGGGGATTCATTAT |
|  | P062-PF | GCACTGCAGATGAGTCGCATAATAA |
|  | P062-KR | ACAGGTACCCATACTTTGTGCAACA |
|  | P063-PF | TCACTGCAGATGAGTTGGATTGAAC |
|  | P063-KR | ATAGGTACCCACGGCGCAAGCACCT |
|  | P064-PF | TTTCTGCAGATGGCGTACGACCTTT |
|  | P064-KR | ATAGGTACCCAATCCAACTCACTCA |
|  | P065-SF | GTCACTAGTATGACGACGCTTCATAAC |
|  | P065-KR | ATAGGTACCCAGAAAGGTCGTACGCC |
|  | P066-PF | TTACTGCAGATGCTTATCGATGCCA |
|  | P066-KR | TATGGTACCCAGTGACTAAAAACGC |
|  | P067-PF | TAGCTGCAGATGCTCTCACTAGATC |
|  | P067-XR | TAATCTAGACATTGGGTCAGCGTCT |
|  | P068-PF | TTACTGCAGATGATACGCCGCCTG |
|  | P068-KR | ATAGGTACCCAGGCGTTCCTGTGAT |
|  | P069-PF | GCCCTGCAGATGAATAAAATCACCA |
|  | P069-KR | TTAGGTACCCATTCTTCAGCCTCCC |
|  | P070-SF | GCCACTAGTATGAGTTTGTTAACCTTG |
|  | P070-KR | TTAGGTACCCATGAAATCGTAACCT |
|  | P071-PF | TTACTGCAGATGATCCAGTTACCGG |
|  | P071-KR | TAAGGTACCCACCCTCCGTAGCTAA |
|  | P072-PF | TTACTGCAGATGAGTCAAGGTGACA |
|  | P072-KR | AATGGTACCCATCTTATGCCTTGTA |
|  | P073-SF | TCTGCATGCATGATAGCGGATTTAA |
|  | P073-KR | AAAGGTACCCACTTCTCCAAAACAG |
|  | P074-PF | AAACTGCAGATGAGCGGAGAAAAGA |
|  | P074-KR | GGTGGTACCCATAACATTTCGGAAT |
|  | P0075-PF | GGGCTGCAGATGAGTCGTATTATAG |
|  | P0075-KR | TTCGGTACCCATCTGGTATTAGGTAACT |
|  | P0076-PF | GAACTGCAGATGGCATCACTAGAGA |
|  | P0076-KR | TAAGGTACCCAGCCTGTGGTTGCTA |
|  | P077-PF | TTACTGCAGATGAGCCAAATTTCAA |
|  | P077-KR | GTCGGTACCCAATAAACCAGATAAG |
|  | P078-PF | GGGCTGCAGATGCAAAATTTACTAA |
|  | P078-KR | ATAGGTACCCAATTCCACCCCACGC |
|  | P079-PF | TTTCTGCAGATGGCTTTTCCGCTAC |
|  | P079-KR | AAAGGTACCCACAATACGCCACGCT |
|  | P080-SF | GGCACTAGTATGAGTTGGGTCTGTCG |
|  | P080-KR | AATGGTACCCATCGAGGTTTACCTC |
|  | P081-PF | TCGCTGCAGATGACACAATTAGAGG |
|  | P081-KR | ATCGGTACCCATTTTAGGTCTCCTG |
|  | P082-PF | TCCCTGCAGATGAGTAACTTCTCTG |
|  | P082-KR | TTAGGTACCCATGGGAACTTCTGTA |
|  | P083-PF | GGGCTGCAGATGAAATATAAACTCA |
|  | P083-KR | GCTGGTACCCATGTTTTTAGTTGCT |
|  | P084-PF | TTTCTGCAGATGACGGTTACCCTTA |
|  | P084-KR | GCTGGTACCCATGTATCCATATCAA |
|  | P085-PF | AAACTGCAGATGCCGAACATAGAAA |
|  | P085-KR | TATGGTACCCACCCCCCCTTCGACA |
|  | P086-PF | GGGCTGCAGATGAAAGTTAAGACTT |
|  | P086-KR | AGAGGTACCCACTTCTTGCGTTGTA |
|  | P087-PF | TTACTGCAGATGGTTACAACGCAAG |
|  | P087-KR | AGAGGTACCCATTTGAACAAATGGC |
|  | P088-PF | TTACTGCAGATGCAGCCATTTGTTC |
|  | P088-KR | CGTGGTACCCATTCTGTAACTTTCA |
|  | P089-PF | GCGCTGCAGATGAAAATCAATACTC |
|  | P089-KR | ATTGGTACCCAGCCGTCAGCCGCCG |
|  | P094-PF | GGGCTGCAGATGAACTTATCATTAA |
|  | P094-BR | CCCGGATCCCAGCTATTTAATAATG |
|  | P097-PF | AAACTGCAGATGCAGAACGCACAAA |
|  | P097-KR | AAAGGTACCCATTTGGCGCAGTTAT |
|  | P098-PF | TTACTGCAGATGATCGGACCAATAT |
|  | P098-KR | GCTGGTACCCATACTTTGAGAAGTG |
|  | P099-PF | AAGCTGCAGATGGGAATTTTTGTTC |
|  | P099-KR | GCCGGTACCCATTTATCCTTATTCA |
